# NFIA differentially controls adipogenic and myogenic gene program through distinct pathways to ensure brown and beige adipocyte differentiation

**DOI:** 10.1371/journal.pgen.1009044

**Published:** 2020-09-29

**Authors:** Yuta Hiraike, Hironori Waki, Kana Miyake, Takahito Wada, Misato Oguchi, Kaede Saito, Shuichi Tsutsumi, Hiroyuki Aburatani, Toshimasa Yamauchi, Takashi Kadowaki

**Affiliations:** 1 Department of Diabetes and Metabolic Diseases, Graduate School of Medicine, The University of Tokyo, Tokyo, Japan; 2 Genome Science Division, Research Center for Advanced Science and Technology, The University of Tokyo, Tokyo, Japan; 3 Department of Diabetes and Lifestyle-Related diseases, Graduate School of Medicine, The University of Tokyo, Tokyo, Japan; 4 Toranomon Hospital, Tokyo, Japan; University of North Carolina, UNITED STATES

## Abstract

The transcription factor nuclear factor I-A (NFIA) is a regulator of brown adipocyte differentiation. Here we show that the C-terminal 17 amino acid residues of NFIA (which we call pro#3 domain) are required for the transcriptional activity of NFIA. Full-length NFIA—but not deletion mutant lacking pro#3 domain—rescued impaired expression of PPARγ, the master transcriptional regulator of adipogenesis and impaired adipocyte differentiation in NFIA-knockout cells. Mechanistically, the ability of NFIA to penetrate chromatin and bind to the crucial *Pparg* enhancer is mediated through pro#3 domain. However, the deletion mutant still binds to *Myod1* enhancer to repress expression of MyoD, the master transcriptional regulator of myogenesis as well as proximally transcribed non-coding RNA called ^*DRR*^*eRNA*, via competition with KLF5 in terms of enhancer binding, leading to suppression of myogenic gene program. Therefore, the negative effect of NFIA on the myogenic gene program is, at least partly, independent of the positive effect on PPARγ expression and its downstream adipogenic gene program. These results uncover multiple ways of action of NFIA to ensure optimal regulation of brown and beige adipocyte differentiation.

## Author summary

Obesity and its complications including type 2 diabetes are growing concerns worldwide. While white adipocytes generally store energy in the form of lipid, classical brown and cold- or β-adrenergic stimulation-induced beige adipocytes dissipate chemical energy in the form of heat through uncoupling protein-1 (Ucp1). Since the re-discovery of human brown and beige adipocytes, it has been considered a promising target for the treatment of obesity. During mesenchymal development, not only activation of brown/beige adipocyte gene program but also repression of muscle gene program is required to achieve thermogenic adipocyte differentiation. Previously, we identified the transcription factor nuclear factor I-A (NFIA) as a regulator of brown adipocyte differentiation. Here we show that the C-terminal 17 amino acid residues of NFIA, which we call pro#3 domain, is required for activation of adipocyte differentiation. However, the deletion mutant which lacks this domain is still able to suppress muscle gene program by repressing the expression of *Myod1*, which encode the master transcriptional regulator of myogenesis, MyoD. We demonstrate that NFIA activates adipogenesis and also “actively” suppresses myogenesis through distinct molecular pathways to ensure brown and beige adipocyte differentiation.

## Introduction

Brown and beige adipose tissues are highly anticipated as a potential target in the treatment of obesity and its complications—including type 2 diabetes. Classical brown adipose tissue (BAT) and cold- or β-adrenergic stimulation-induced beige adipose tissue dissipate chemical energy in the form of heat through uncoupling protein-1 (Ucp1) on the mitochondrial inner membrane, while white adipose tissue (WAT) generally stores energy in the form of lipid. Beyond heat generation, the physiological functions of brown and beige fat—including endocrine function—are currently being thoroughly investigated [1].

Previously, we had identified a transcription factor NFIA (nuclear factor I-A) as a transcriptional regulator of brown fat by a genome-wide open chromatin analysis of murine brown fat and by subsequent comprehensive functional analyses [2]. NFIA binds to and activates the brown-fat-specific enhancers even before differentiation and later facilitates the binding of PPARγ (peroxisome proliferator-activated receptor γ—a master transcription factor of adipogenesis), to control the brown fat gene program. The brown fat of NFIA-KO mouse neonate showed impaired expression of the brown fat gene program and a reciprocal elevation of the muscle gene program. In human brown and beige adipose tissue, expression levels of *NFIA* and the brown-fat-specific genes including *UCP1* are positively correlated.

NFIA has at least three functions on the transcriptional regulation of brown fat [2]. First, NFIA activates adipogenesis per se, through activating the transcription of *Pparg*, which encodes PPARγ. Second, NFIA also activates the brown-fat-specific gene expression (such as *Ucp1* and *Ppargc1a*) independent of the degree of adipocyte differentiation, through facilitating the binding of PPARγ to the brown-fat-specific enhancers. Third, NFIA represses myogenesis through suppression of myogenic transcription factors such as *Myod1* as well as *Myog*, while its mechanism(s) has been less well characterized. To date, functional domains responsible for these three functions remain elusive. To address this question, here we performed a site-directed mutagenesis to construct a vector expressing various deletion mutants of NFIA, based on the previous literature about the transcriptional activity of NFIC [3,4]. By integrating cellular experiments using these deletion mutants, RNA-seq, FAIRE-seq (formaldehyde assisted isolation of regulatory elements coupled with high-throughput sequencing), ChIP-seq (chromatin immunoprecipitation coupled with high-throughput sequencing) analysis and a CRISPR dCas9-Krab mediated epigenome editing experiment, we ascertained that a C-terminal proline rich domain of NFIA, especially the C-terminal 17 amino acid residues of NFIA (aa 493–509), which we call pro#3 domain here, is crucial for inducing an adipocyte-specific *Pparg2* expression and adipogenesis. Using Cre-mediated knock out (KO) of NFIA in primary adipocytes derived from *Nfia*^flox/flox^ mice, we demonstrated that this domain is indispensable in order to rescue impaired beige adipogenesis caused by NFIA-KO. However, strikingly, this domain is dispensable for suppressing myogenesis. Both full-length NFIA and mutant NFIA that lacks pro#3 domain (Δpro#3 mutant) can suppress expression of *Myod1*, a master transcription factor of myogenesis and its downstream effector *Myog*, by binding to the enhancer of *Myod1* and its proximally located enhancer RNA, called ^*DRR*^*eRNA*, which activates the *Myog* transcription *in trans* [5]. PPARγ and MyoD, the master transcriptional regulator of adipogenesis and myogenesis, respectively, antagonize each other to ensure mutually exclusive, discrete cell fate decisions [6]. These results thus indicate that NFIA suppresses the myogenic gene program by both the PPARγ-dependent pathway mediated by the C-terminal pro#3 domain, and the PPARγ-independent pathway which does not require a pro#3 domain, to ensure adipocyte differentiation.

## Results

### The C-terminal proline rich domain is required for the transcriptional activity of NFIA

**Fig 1:**
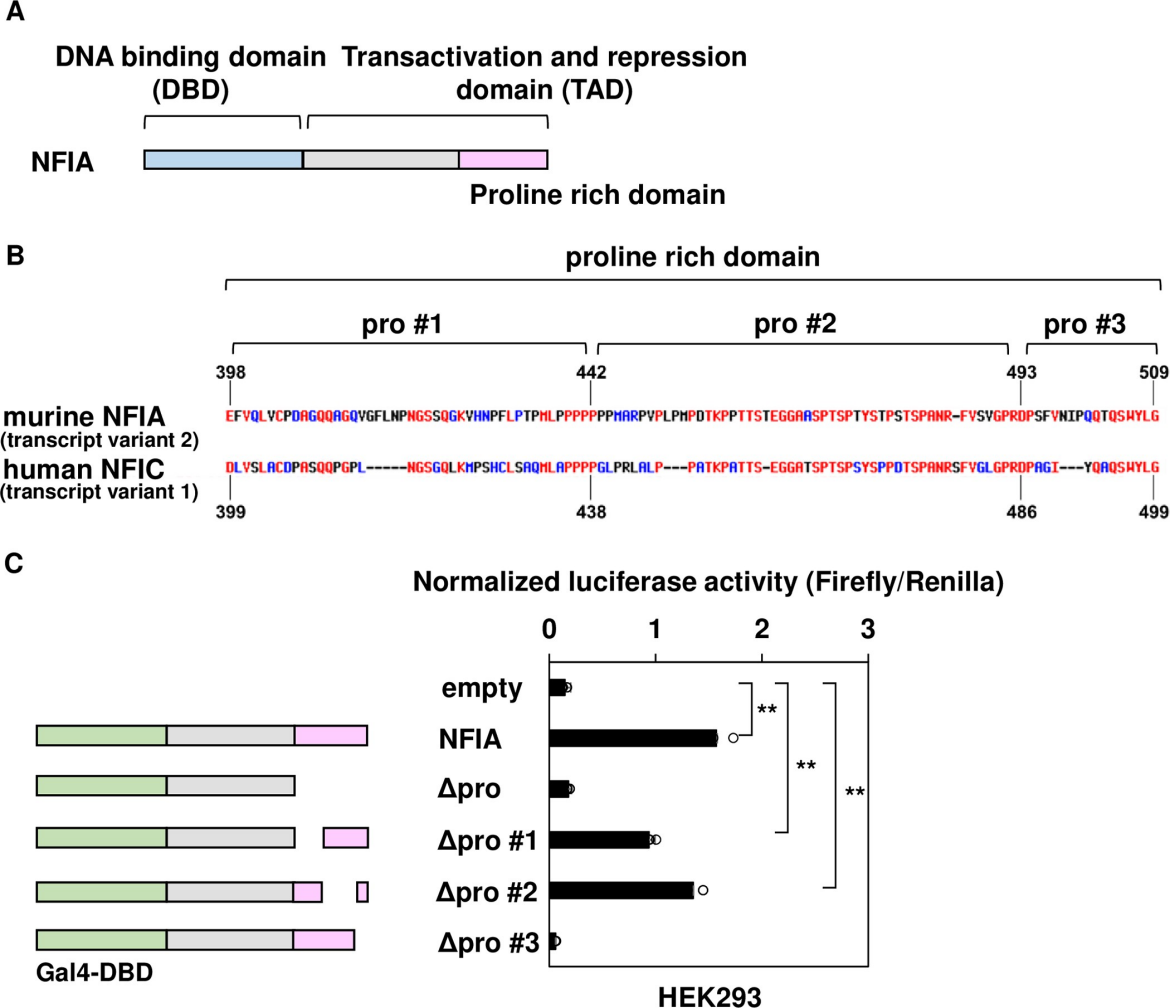
The C-terminal proline rich domain is required for the transcriptional activity of NFIA. (A) A diagram showing the domain structure of NFIA. DNA binding domain (DBD) is shown in blue, proline-rich domain is shown in pink, and transactivation domain (TAD) is shown in gray and pink combined. (B) A detailed diagram showing the C-terminal proline rich domain of murine NFIA, variant 2 as well as human NFIC, variant 1. (C) Transcriptional activities of full-length and mutant NFIA examined by luciferase reporter assay. (mean +/- S.E.M.; N = 4; ** *P* <0.01).

There are four isoforms of NFI in vertebrates (NFIA, B, C and X) and they are expressed in a unique but overlapping manner during development [7]. The structure of transcription factors generally consists of a DNA-binding domain (DBD) that directly recognizes specific DNA sequences and binds to them, and a transactivation domain (TAD) that is responsible for the interaction with other proteins—including transcriptional co-regulators. All isoforms of NFI, including NFIA, have highly conserved DBD in their N-terminal region, and TAD in their C-terminal region (Fig 1A). In case of human NFIC, among its TAD, the C-terminal proline rich domain is crucial for its transcriptional activity [3,4]. While we did not find substantial sequence similarity in proline rich domain between murine NFIA and human NFIC compared to other domains (overall: 63%, DBD: 91%, TAD outside of proline rich domain: 49%, proline rich domain: 49%, S1A Fig), we did find evolutionally conservation in proline rich domain of NFIA from *Homo sapiens*, *Mus musculus*, *Gallus gallus domesticus* to *Danio rerio* (*Homo sapiens* vs *Danio rerio*, overall: 85%, DBD: 95%, TAD outside of proline rich domain: 71%, proline rich domain: 92%, S1B Fig). This observation prompted us to examine the possible role of the C-terminal proline rich domain in the transcriptional activity of NFIA. We constructed a vector expressing fusion protein of yeast-derived Gal4 DBD and either full-length or mutant NFIA TAD to examine their activity to transactivate an MH100-*Tk*-luciferase reporter gene expression through GAL4 binding elements. This experiment clearly showed that Δpro mutant (which lacks the proline rich domain of NFIA that is depicted in Fig 1B) lacked the ability to activate the reporter gene expression. More specifically, while Δpro#1 and Δpro#2 mutant showed substantial activity, Δpro#3 mutant completely lacked the effect, suggesting that a crucial region for the transcriptional activity of NFIA lies within pro#3 domain (Fig 1C).

### The pro#3 domain of NFIA is indispensable in driving adipogenesis

**Fig 2:**
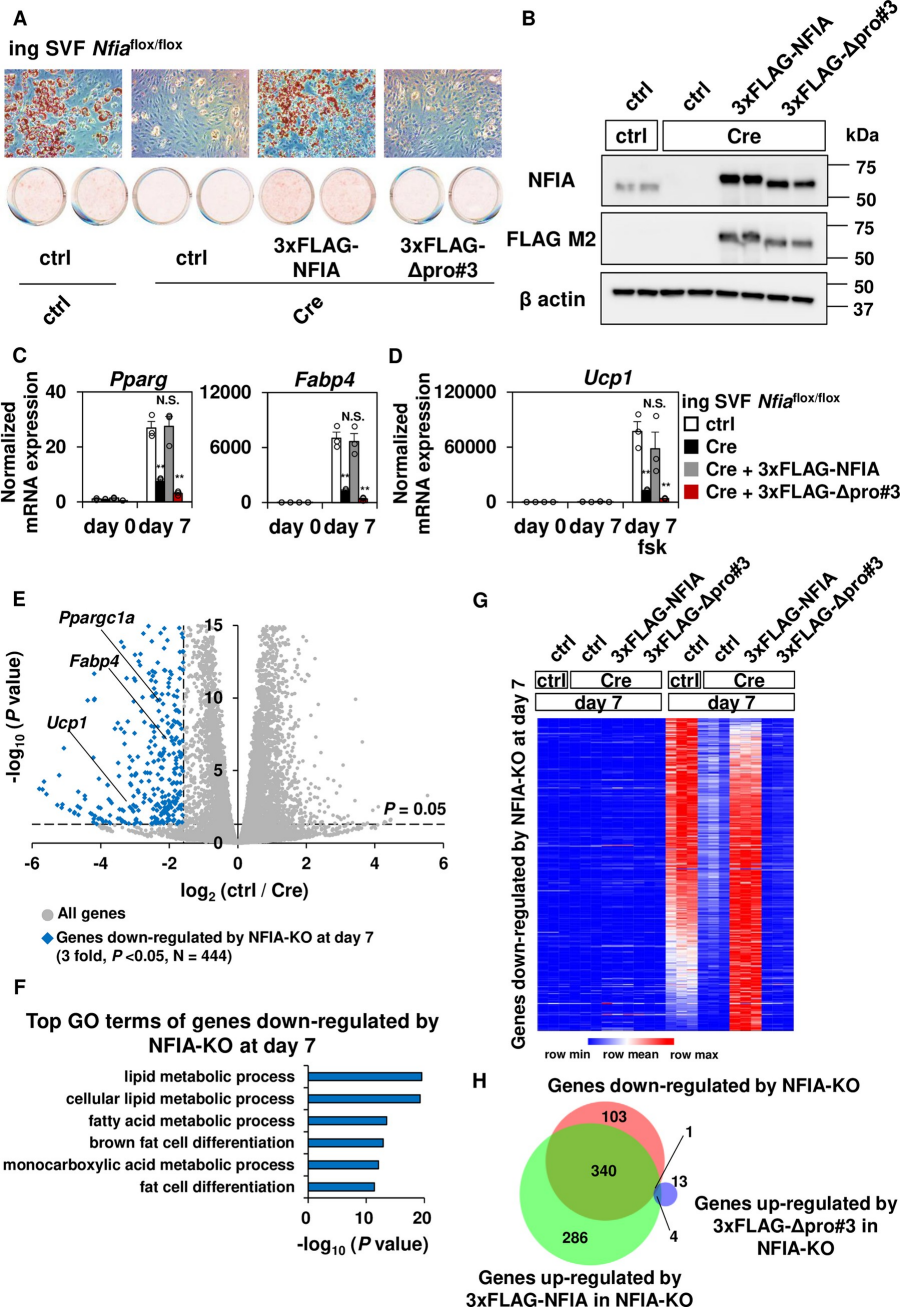
The pro#3 domain of NFIA is indispensable for driving adipogenesis. (A) Control cells, Cre-expressing cells and cells expressing cre as well as either full-length NFIA or Δpro#3 mutant were stained with Oil-Red-O seven days after inducing adipocyte differentiation. (B) Western blot analysis of endogenous NFIA as well as overexpressed, FLAG-tagged NFIA protein expression in indicated cells. β-actin was used as a loading control. (C-D), Common adipocyte genes (C) and the brown-fat-specific genes (D) were quantified by RT-qPCR at the indicated time course (mean +/- S.E.M.; N = 3; * *P* <0.05, ** *P* <0.01). When indicated, forskolin (fsk) treatment was performed to increase intracellular cyclic AMP levels. (E) Volcano plot of RNA-seq analysis of ctrl cells as well as Cre-mediated NFIA-KO cells at day 7 of differentiation. Genes significantly down-regulated by NFIA-KO, determined by DeSeq2, are shown in blue (3 fold, *P* <0.05, N = 444). RNA-seq was performed in triplicate for each condition. (F) Gene ontology analysis of genes down-regulated by NFIA-KO at day 7 of differentiation. (G) Heatmap representation of genes down-regulated by NFIA-KO at day 7, before and after differentiation. Note that pseudo-count of FPKM 1 was added to all FPKM values to decrease the effect of noise of low-expressed genes. Row normalized (FPKM+1) was depicted in heat map after hierarchical clustering. RNA-seq was performed in triplicate for each condition. (H) Venn diagram showing the overlap of genes down-regulated by NFIA-KO, genes up-regulated by 3xFLAG-NFIA in NFIA-KO, and genes up-regulated by 3xFLAG-Δpro#3 mutant. Significantly up- or down-regulated genes were determined by DeSeq2 (3 fold, *P* < 0.05).

To test the role of pro#3 domain for the effect of NFIA on adipocyte differentiation in physiological context, we attempted to examine whether overexpression of Δpro#3 mutant can rescue the effect of knocking out of NFIA in primary adipocytes. First, we isolated the stromal vascular fraction (SVF) from inguinal white adipose tissue of *Nfia*^flox/flox^ mice (S2A Fig) and immortalized the cells using SV40 large T antigen. Then we infected them with either empty or Cre recombinase-expressing retroviral vector, and induced adipocyte differentiation using the cocktail for thermogenic adipocytes (S2B Fig). Cre-mediated recombination was efficiently induced when judged by the PCR of genomic DNA using the primer pair that flanks the *lox P* site (S2C Fig), and expression of NFIA was almost completely ablated at both the mRNA (S2D Fig) and protein levels (S2E Fig) in Cre-expressing cells. While introduction of Cre recombinase into wild-type SVF did not affect mRNA expression of *Nfia* and *Fabp4* (S2I Fig), Cre-mediated knock out of NFIA in *Nfia*^flox/flox^ mice-derived SVF resulted in severely impaired adipocyte differentiation as evaluated by the degree of lipid accumulation as well as mRNA expression of the *Pparg* and its target *Fabp4* (S2B and S2F Fig). Accordingly, mRNA expression of *Ppara* as well as *Ppargc1a* and *Ucp1* in response to elevated cyclic AMP through forskolin (fsk) treatment was also severely reduced in Cre-expressing cells (S2G and S2H Fig). These results indicate that NFIA is required for thermogenic adipocyte differentiation in a cell-autonomous manner. Second, we performed a rescue experiment to examine whether Δpro#3 mutant can restore the impaired adipocyte differentiation observed in Cre-expressing cells (Fig 2A). While overexpressed full-length NFIA and Δpro#3 mutant were expressed at the similar levels (Fig 2B and S3A Fig), Δpro#3 mutant failed to restore the impaired adipocyte differentiation (Fig 2A and 2C) and the brown-fat-specific gene expression such as *Ppara*, *Ppargc1a* and *Ucp1* in Cre-expressing cells (Fig 2D and S3C Fig). Of note, we used *Nfia* transcript variant 2 (NM_010905.3, uc008ttu.2), which is the most abundantly expressed isoform of *Nfia* in adipocytes, for gain of function studies (S3E, S3F and S3G Fig) and changes in mRNA expression of *Nfib*, *Nfic* and *Nfix* upon knocking out of NFIA and introduction of full-length NFIA as well as the Δpro#3 mutant were modest if compared to the expression levels of *Nfia* itself (S3B Fig).

We next performed RNA-seq analysis of these four cell types (ctrl, Cre-mediated NFIA -KO, NFIA-KO + 3xFLAG-NFIA, NFIA-KO + 3xFLAG-Δpro#3) before and after differentiation. Unsupervised hierarchical clustering of gene expression after differentiation revealed seven cluster of genes, characterized by distinct gene ontology (GO) terms (cluster A to G, S4B–S4I Fig). Knocking out of NFIA resulted in down-regulation of genes in cluster F, which is characterized by GO terms related to lipid metabolism, and the down-regulation was restored by expression of full-length NFIA but not by Δpro#3 mutant (S4B and S4H Fig). Genes in cluster E, which were characterized by similar GO terms although significance of enrichment was much weaker than that of cluster F, were relatively unchanged upon NFIA KO but up-regulated by introduction of full-length NFIA (S4B and S4G Fig). On the other hand, knocking out of NFIA resulted in de-repression of genes in cluster B slightly and also genes in cluster C strongly, both characterized by GO terms like adhesion. Introduction of full-length NFIA, but not Δpro#3 mutant, restored the repression (S4B, S4D and S4E Fig).

When we independently defined genes down-regulated by NFIA-KO in differentiated adipocytes using DeSeq2 (Fig 2E, three fold, *P* < 0.05, N = 444), these genes are characterized by GO terms related to lipid metabolism and brown fat cell differentiation (Fig 2F). The heat map representation of genes down-regulated by NFIA-KO, including the four cell types before and after differentiation (Fig 2G), clearly indicated that introduction of full-length NFIA but not the Δpro#3 mutant is able to restore expression of genes that were down-regulated by NFIA-KO, and most of the genes down-regulated by NFIA-KO are induced during adipocyte differentiation. Consistently, genes down-regulated by NFIA-KO and genes up-regulated by 3xFLAG-NFIA in NFIA-KO, but not genes up-regulated by 3xFLAG-Δpro#3 significantly overlapped each other (Fig 2H). Likewise, genes up-regulated by NFIA-KO substantially overlapped only with genes down-regulated by 3xFLAG-NFIA in NFIA-KO, but not with genes down-regulated by 3xFLAG—Δpro#3 in NFIA-KO (S4A Fig). Together, these results suggest that a pro#3 domain is required for NFIA to induce thermogenic adipocyte differentiation.

### The NFIA Δpro#3 mutant retains the ability to suppress myogenesis

**Fig 3:**
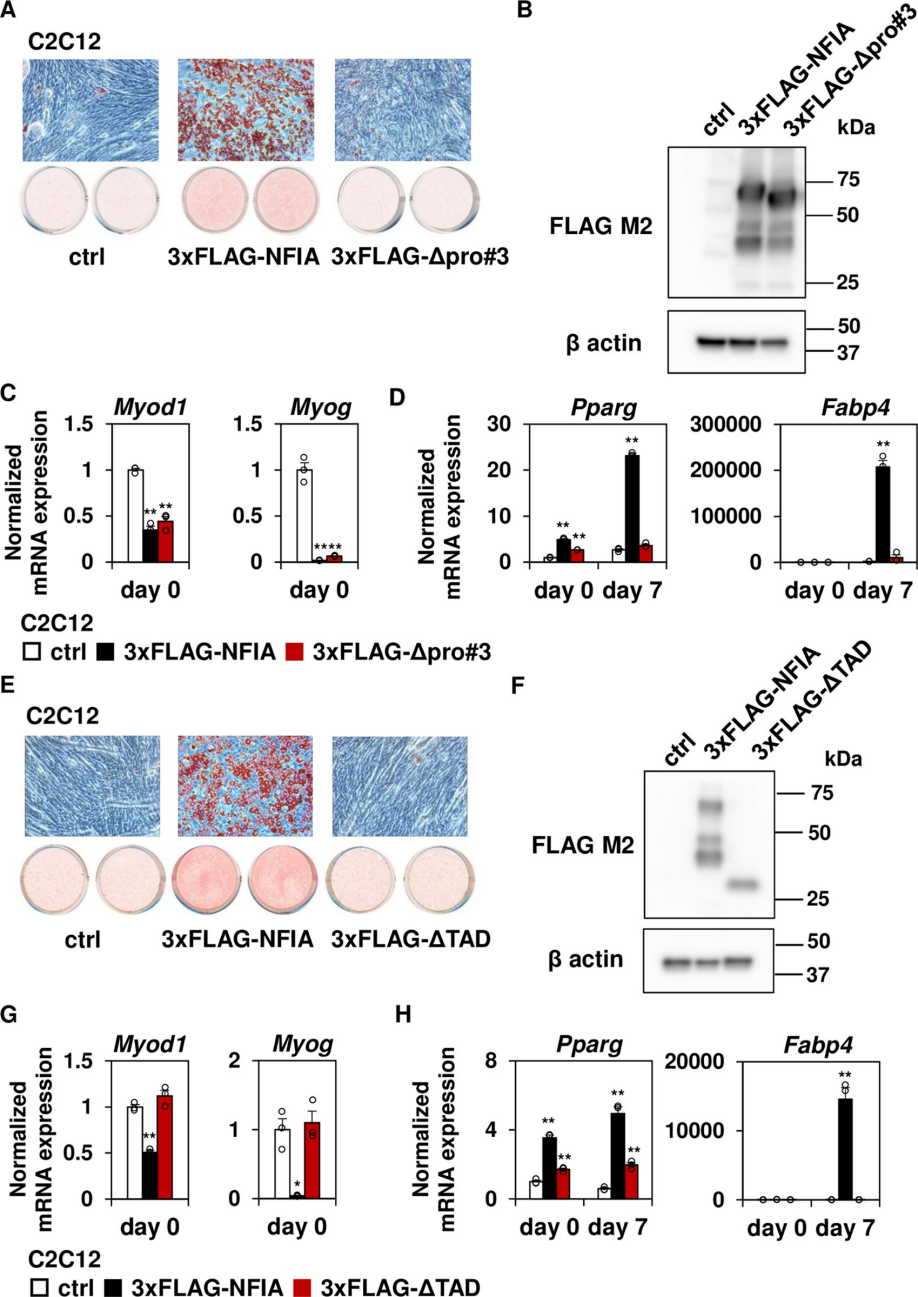
The NFIA Δpro#3 mutant, but not ΔTAD mutant suppresses myogenesis. (A) Control-, full-length NFIA- and the Δpro#3 mutant-expressing cells were stained with Oil-Red-O seven days after inducing adipocyte differentiation. (B)Western blot analysis using FLAG M2 antibody in cells expressing 3xFLAG-tagged full-length NFIA and the Δpro#3 mutant. β-actin was used as a loading control. (C-D) Myogenic genes (C) and adipocyte genes (D) were quantified by RT-qPCR at the indicated time course (mean +/- S.E.M.; N = 3; * *P* <0.05, ** *P* <0.01). (E) Control-, full-length NFIA- as well as ΔTAD mutant-expressing cells were stained with Oil-Red-O seven days after inducing adipocyte differentiation. (F) Western blot analysis using FLAG M2 antibody in cells expressing 3xFLAG-tagged full-length NFIA and ΔTAD mutant. β-actin was used as a loading control. (G-H) Myogenic genes (G) and adipocyte genes (H) were quantified by RT-qPCR at the indicated time course (mean +/- S.E.M.; N = 3; * *P* <0.05, ** *P* <0.01).

During mesenchymal development, not only activation of the adipocyte gene program but also repression of the muscle gene program is required to achieve adipocyte differentiation. In this context, we next introduced full-length NFIA as well as Δpro#3 mutant into C2C12 myoblast cell lines to examine whether they can repress the muscle gene program. Introduction of full-length NFIA (but not Δpro#3 mutant) resulted in lipid accumulation as evaluated by Oil-Red-O staining (Fig 3A). Then we confirmed protein expression of 3xFLAG-tagged full length NFIA as well as Δpro#3 mutant (Fig 3B). Quite notably, while Δpro#3 mutant failed to induce adipocyte differentiation (Fig 3A and 3D), this mutant still retained the ability to suppress muscle gene expression including *Myod1*, the master transcriptional regulator of myogenesis and its downstream effector *Myog* (Fig 3C). Consistently, introduction of full-length NFIA as well as the Δpro#3 mutant tended to repress *Myog* expression and significantly repressed *Myl9* expression in NFIA-KO preadipocytes, suggesting that NFIA is able to repress myogenic genes in preadipocytes and that the pro#3 domain is dispensable for this function (S3D Fig). Moreover, a mutant lacking whole TAD (ΔTAD mutant, Fig 3F) was not only failed to induce both lipid accumulation (Fig 3E) and adipocyte gene expression (Fig 3H) but also failed to repress myogenic gene expression (Fig 3G), suggesting that that activation of adipogenesis and suppression of myogenesis is mediated, at least in part, by distinct functional domains of NFIA and that the region responsible for suppressing myogenesis lies outside of the pro#3 domain.

**Fig 4:**
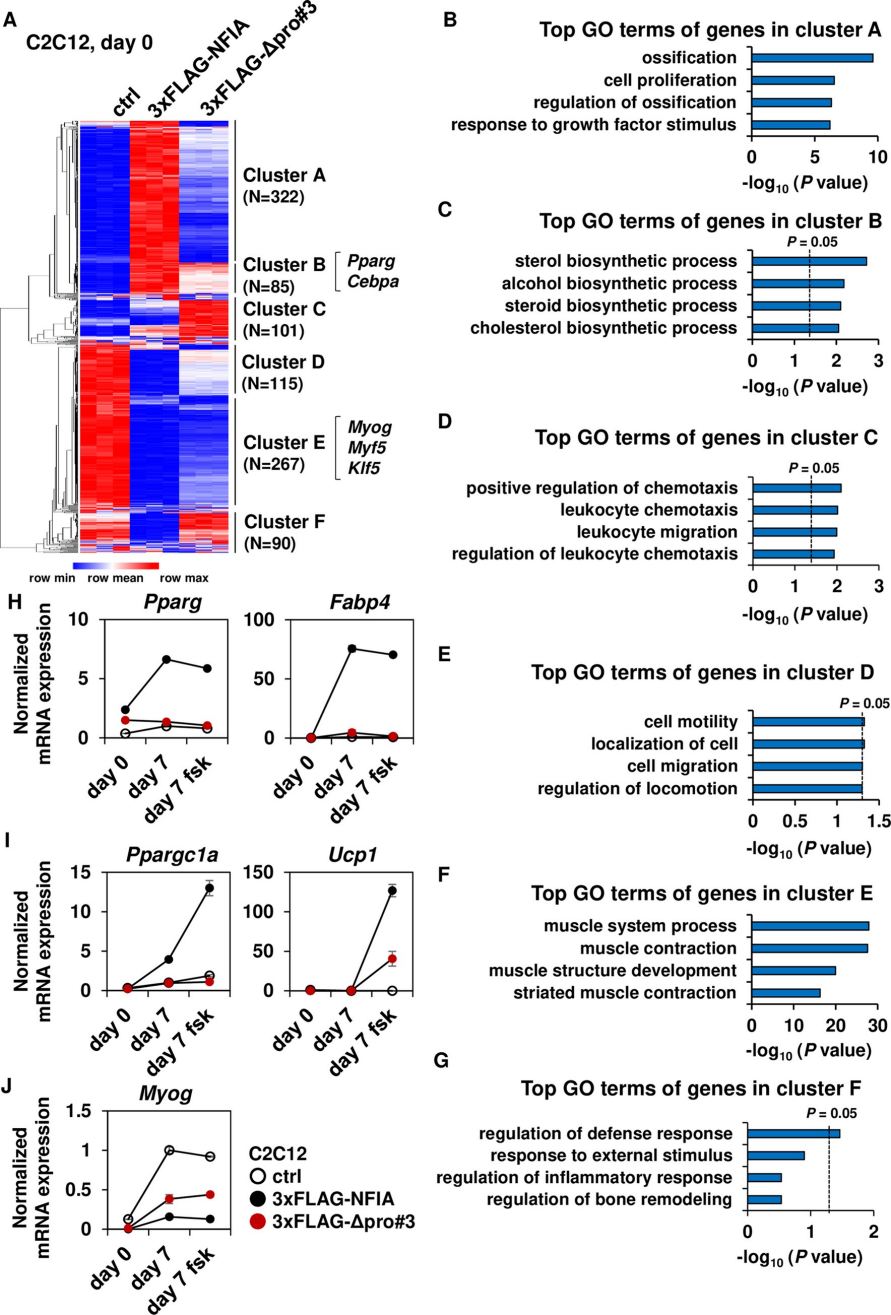
The NFIA Δpro#3 has the ability to suppress muscle gene program even when examined in a genome-wide manner. (A) A heat map representation of genes with greater than three fold changes in expression (N = 980). Note that pseudo-count of FPKM 1 was added to all FPKM values to decrease the effect of noise of low-expressed genes. Row normalized (FPKM+1) was depicted in heat map after hierarchical clustering. RNA-seq was performed in triplicate for each condition. (B-G), Top GO terms of genes in cluster A-D, as defined in (A). (H-J), Normalized mRNA expression during adipocyte differentiation of common adipocyte (H), brown-fat-specific genes (I) and muscle specific genes (J) (mean +/- S.E.M.; N = 3). When indicated, forskolin (fsk) treatment was performed to increase intracellular cyclic AMP levels.

To gain further insight on genome-wide changes in gene expression mediated by introduction of full-length NFIA as well as Δpro#3 mutant into C2C12 cells, we performed RNA-seq analysis of control, full-length NFIA- and Δpro#3-expressing cells before and after differentiation. While NFIA is known to heterodimerize with NFIB, C and X [7], changes in the expression patterns of *Nfib*, *Nfic* and *Nfix* upon introduction of full-length NFIA as well as the Δpro#3 mutant are modest if compared to the expression levels of retrovirally introduced NFIA (S5A Fig), suggesting that the contribution of the heterodimerization is limited, at least in this experiment. Unsupervised hierarchical clustering of differentially expressed genes before differentiation showed six distinct gene expression patterns, namely cluster A to F (Fig 4A). The heat map representation of clustering analysis clearly showed that Δpro#3 mutant cannot adequately activate genes up-regulated by full-length NFIA (cluster A, N = 322 and cluster B, N = 85), while Δpro#3 mutant can still repress genes down-regulated by full-length NFIA (cluster D, N = 115 and cluster E, N = 267) in a genome-wide manner. Gene ontology (GO) analysis of each gene cluster (Fig 4B–4G) demonstrated that genes in cluster E were entirely characterized by GO terms related to muscle development and function (Fig 4F), strongly suggesting that majority of the genes down-regulated by both full-length NFIA and Δpro#3 mutant are related to myogenesis. Although genes in cluster B were not necessarily enriched by GO terms related to adipocyte differentiation, this gene cluster included transcription factors reported to be crucial for adipogenesis including *Pparg*, *Cebpa* and *Zfp423*. Indeed, expression of *Pparg* and its target *Fabp4* were strongly induced by full-length NFIA during adipocyte differentiation (Fig 4H), and this was also the case for the brown-fat-specific genes including *Cidea*, *Ppargc1a* and *Ucp1* especially after forskolin (fsk) treatment to increase intracellular cyclic AMP levels (Fig 4I and S5B Fig). On the other hand, myogenic genes such as *Myog* and its target *Myl4* and *Myh8* were strongly suppressed by full-length NFIA throughout the differentiation; they were also suppressed by Δpro#3 mutant although to a lesser extent (Fig 4J and S5C Fig). Interestingly, genes in cluster C that are characterized by GO terms like leukocyte chemotaxis, were up-regulated only by Δpro#3 mutant (Fig 4D), suggesting that pro#3 domain is required for down-regulation of these immune-related genes.

We also performed hierarchical clustering of gene expression in cells after differentiation (S6 Fig) and this analysis showed that genes up-regulated by full-length NFIA but not by Δpro#3 mutant were characterized by GO terms like lipid metabolism and fat cell differentiation (cluster A, S6A and S6B Fig), and genes down-regulated by both full-length NFIA and Δpro#3 mutant were related to GO terms like muscle cell differentiation (cluster E, S6A and S6F Fig). Collectively, these results indicate that Δpro#3 mutant can suppress the muscle gene program throughout the differentiation even when examined in a genome-wide manner.

### Genome-wide landscape of NFIA binding and changes in chromatin accessibility

**Fig 5:**
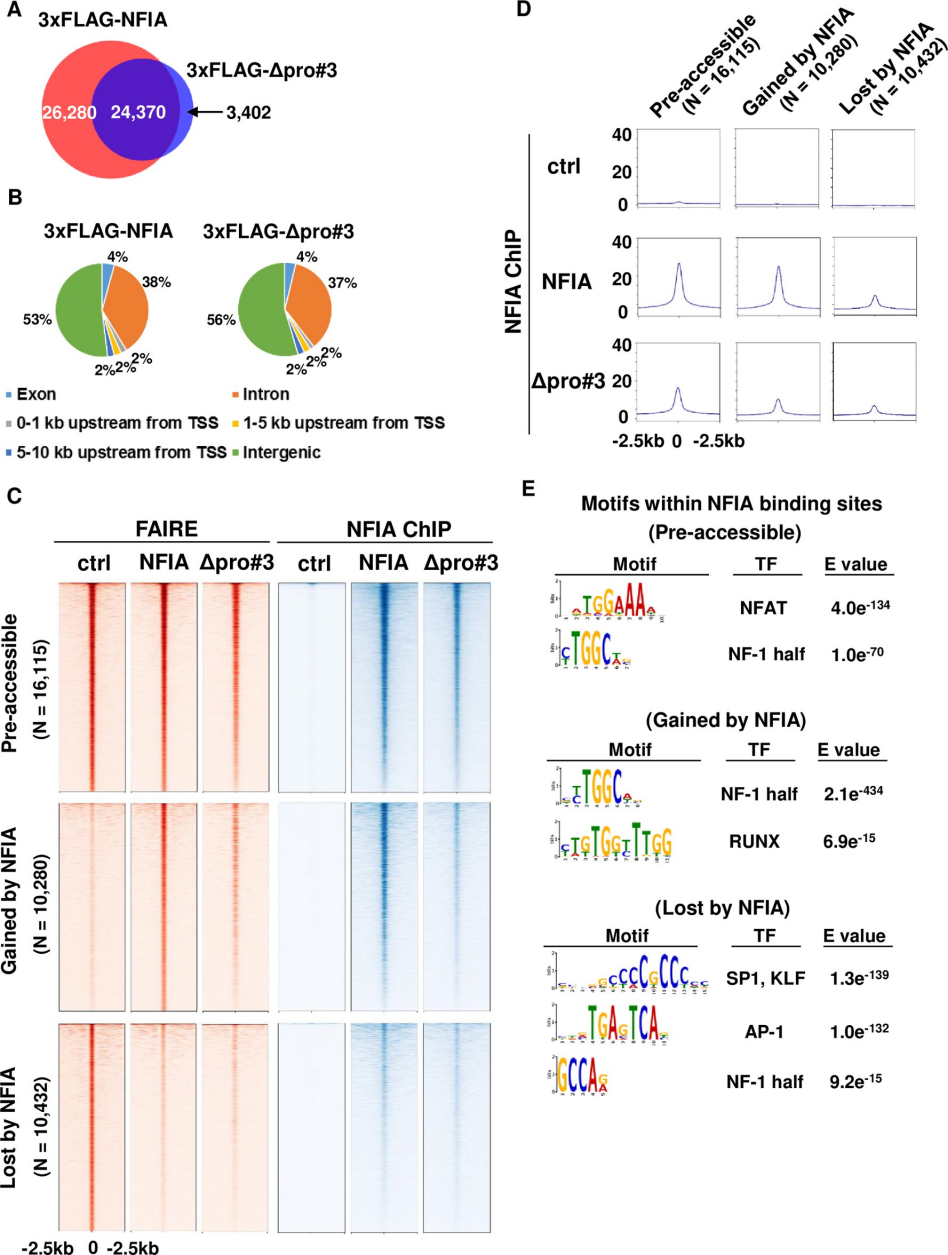
Genome-wide landscape of NFIA binding and changes in chromatin accessibility. (A) Venn diagram showing the overlap of full-length NFIA and Δpro#3 mutant at day 0 of differentiation. (B) Genomic location of full-length NFIA and Δpro#3 mutant at day 0 of differentiation. (C) A heat map representation showing tag density signals of FAIRE and NFIA ChIP in indicated cells at pre-accessible chromatin sites in control cells that are still accessible by introduction of full-length NFIA (“Pre-accessible”, N = 16,115), full-length NFIA-dependent accessible chromatin sites (“Gained by NFIA”, N = 10,280) and full-length NFIA-dependent inaccessible chromatin sites (“Lost by NFIA”, N = 10,432). (D) An aggregate plot showing tag density signal of NFIA ChIP at the regions shown in (C). (E). *De novo* motif analysis of NFIA binding regions in indicated sites.

We next performed ChIP-seq analysis of both full-length NFIA and the Δpro#3 mutant in C2C12 myoblasts at day 0 of differentiation using 3xFLAG antibody, to identify binding sites of these proteins globally and gain mechanistic insights underlying the changes in gene expression caused by these two proteins. We also performed FAIRE-seq analysis of ctrl, full-length NFIA- and Δpro#3 mutant-expressing cells to identify accessible chromatin regions. Peak calling of the ChIP-seq dataset identified 50,818 binding sites for full-length NFIA and 28,433 sites for Δpro#3 mutant, respectively. These binding sites substantially overlapped each other (Fig 5A), and both of these two proteins preferentially binds to both intergenic and intronic regions (Fig 5B). *De novo* motif analysis of full-length NFIA as well as Δpro#3 mutant recovered motifs similar to previously reported NF-1 motifs [2], a finding consistent with direct binding to the genome (S7A Fig).

To examine the genome-wide landscape of full-length NFIA and Δpro#3 mutant binding—plus the changes in chromatin accessibility caused by them, we first defined pre-accessible chromatin sites in control cells that were still accessible by introduction of full-length NFIA (“Pre-accessible”, N = 16,115), full-length NFIA-dependent accessible chromatin sites (“Gained by NFIA”, N = 10,280) and full-length NFIA-dependent inaccessible chromatin sites (“Lost by NFIA”, N = 10,432). Heat map representation showed that chromatin accessibility of “Gained by NFIA” sites was lower in Δpro#3-expressing cells than that in full-length NFIA-expressing cells, while accessibility of “Lost by NFIA” sites was similarly low in cells expressing full-length NFIA and Δpro#3. And that applied also to the binding signals of these two proteins (Fig 5C). Besides, aggregate plots showed that the binding signal of Δpro#3 mutant in “Gained by NFIA” sites was clearly lower than the signal in “pre-accessible” sites, although the binding signal of full-length NFIA was comparable between these two sites (Fig 5D). Furthermore, both full-length NFIA and Δpro#3 mutant bound to “Lost by NFIA” sites although the binding signals were weaker when compared with the binding to “pre-accessible” or “Gained by NFIA” sites. We next performed *de novo* motif analysis of NFIA binding sites within “Pre-accessible”, “Gained by NFIA”, and “Lost by NFIA” sites, to explore the possibility that different co-localizing or competing factors account for the differential effect of NFIA on chromatin at these sites. As expected, “Gained by NFIA” sites were strongly enriched with NF-1 motif, followed by mild enrichment of RUNX motif. Intriguingly, along with NF-1 motif, “Pre-accessible” sites were enriched with NFAT motif, and “Lost by NFIA” sites were enriched with SP-1, KLF and AP-1 motifs, respectively (Fig 5E). Notably, SP-1 and some members of the KLF family including KLF5, have been reported as a positive regulator of myogenesis [8–10]. These results demonstrate the genome-wide effect of NFIA on both positive and negative regulation of gene expression at the chromatin levels, and suggest that the ability of NFIA to penetrate otherwise inaccessible chromatin and increase accessibility is mediated by pro#3 domain, while the domain is dispensable for negative regulation of chromatin accessibility.

### The NFIA Δpro#3 mutant is unable to penetrate inaccessible chromatin to activate target gene expression

**Fig 6:**
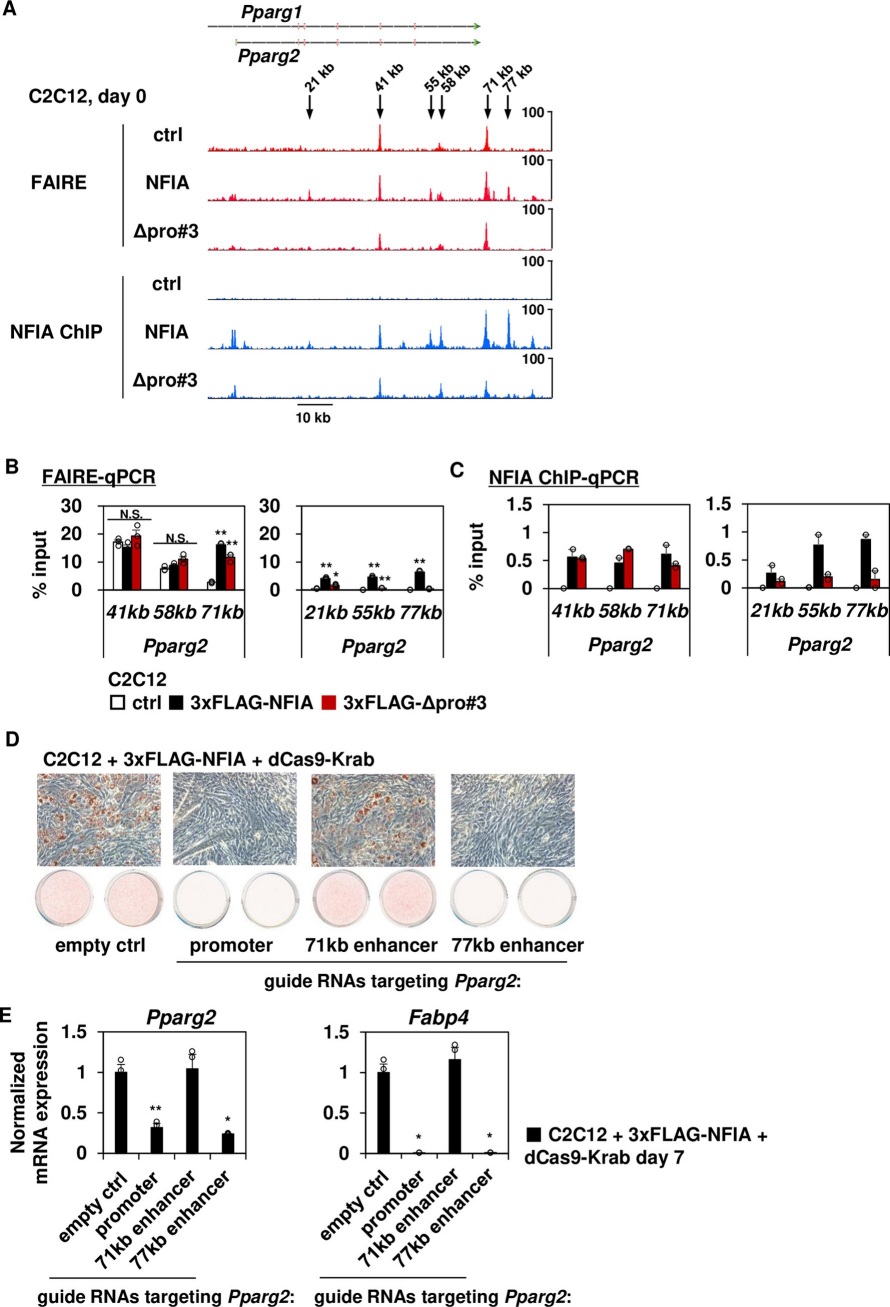
Direct binding of full-length NFIA to the *Pparg2* 77kb enhancer is indispensable for NFIA-driven adipogenesis. (A) FAIRE-seq and NFIA ChIP-seq tracks in indicated cells at day 0 of differentiation at the *Pparg* locus. (B) FAIRE-qPCR analysis of indicated loci (mean +/- S.E.M.; N = 3; * *P* <0.05, ** *P* <0.01). (C) ChIP-qPCR analysis for 3xFLAG-full-length NFIA and 3xFLAG-Δpro#3 mutant of indicated loci using the FLAG M2 antibody. (mean +/- S.E.M.; N = 2). The representative result of multiple independent experiments is shown. (D) NFIA-expressing C2C12 myoblasts with lentivirally introduced dCas9-Krab and indicated guide RNA were stained with Oil-Red-O seven days after inducing adipocyte differentiation. (E) qPCR analysis of indicated genes in NFIA-expressing C2C12 myoblasts with lentivirally introduced dCas9-Krab and indicated guide RNA (mean +/- S.E.M.; N = 3).

To explore the mechanism(s) of pro#3 domain-dependent adipogenic effect of NFIA, we inspected the binding pattern of full-length NFIA as well as Δpro#3 mutant and changes in chromatin accessibility caused by their binding, at the genomic locus near *Pparg* that encodes the master transcriptional regulator of adipogenesis, PPARγ (Fig 6A). When we looked at six genomic regions near *Pparg2* (*Pparg2* 21kb, 41kb, 55kb, 58b, 71kb and 77kb), while pre-accessible regions in ctrl cells can be occupied by both full-length NFIA and Δpro#3 mutant (*Pparg2* 41kb, 58kb, and 71kb), full-length NFIA-dependent accessible regions can be efficiently occupied only by full-length NFIA (*Pparg2* 21kb, 55kb, and 77kb, Fig 6A). Independently-performed FAIRE-qPCR and ChIP-qPCR experiment confirmed this observation (Fig 6B and 6C).

To directly examine the functional requirement of NFIA binding sites near *Pparg* in a loss-of-function manner, we performed a CRISPR dCas9-Krab mediated epigenome editing experiment [11]. Catalytically-inactive Cas9 fused with transcriptional repressor Krüppel-associated box (Krab) domain and locus-specific guide RNA enabled us to examine the requirement of individual NFIA binding sites for driving expression of *Pparg*. First, we examined whether direct binding of NFIA to the promoter of *Pparg2*, the adipocyte-specific isoform of *Pparg* mRNA, which encodes PPARγ2, is required for the effect of NFIA on adipogenesis. Lentiviral introduction of dCas9-Krab along with guide RNA targeting the *Pparg2* promoter resulted in significantly decreased expression of *Pparg2*, while expression levels of *Pparg1* were maintained, in NFIA-expressing C2C12 myoblasts (S7B Fig). Consequently, expression levels of *Fabp4* were significantly decreased (S7B Fig), indicating that direct binding and activation of the *Pparg2* promoter by full-length NFIA is indispensable for its effect on adipogenesis. Next, we examined whether the *Pparg2* 71kb enhancer (pre-accessible site) or the 77kb enhancer (NFIA-dependent accessible site) is required for NFIA-driven adipogenesis. While introduction of dCas9-Krab along with guide RNA targeting the *Pparg2* 71kb enhancer did not affect adipocyte differentiation when evaluated by Oil-Red-O staining as well as expression levels of *Pparg2* and *Fabp4* in NFIA-expressing C2C12 myoblasts, CRISPR dCas9-Krab-mediated suppression of the *Pparg2* 77kb enhancer significantly attenuated lipid droplet formation (Fig 6D) and down-regulated expression of *Pparg2* and *Fabp4* (Fig 6E), suggesting that the *Pparg2* 77kb enhancer, an NFIA-dependent accessible site, is indispensable for NFIA-driven adipogenesis. Moreover, the *Pparg2* 77kb enhancer is also indispensable for adipocyte differentiation in 3T3-F442A cells (S7C Fig), suggesting that this NFIA-dependent accessible enhancer is required for adipogenesis in more physiologically-relevant conditions. Intriguingly, *Pparg2* 77kb enhancer did not exhibit transcriptional activity in luciferase reporter assay when co-introduced with full-length NFIA or the Δpro#3 mutant in HEK293 cells, suggesting that native chromatin contexts including long-range chromatin interaction are required for the *Pparg2* 77kb enhancer to exert its effect (S8A Fig). We also found pro#3 domain-dependent NFIA binding sites near *Cebpa*, a well-known adipogenic transcription factor whose expression was up-regulated by NFIA (S8B Fig). Overall, these results indicate that the ability of NFIA to directly bind to previously inaccessible *Pparg2* enhancer and to increase its accessibility requires pro#3 domain, and *Pparg2* 77kb enhancer is indispensable for NFIA to activate adipogenesis through increasing the *Pparg2* expression.

### NFIA directly suppresses expression of *Myod1* and ^*DRR*^*eRNA*, and also suppresses that of *Myog in trans*

**Fig 7:**
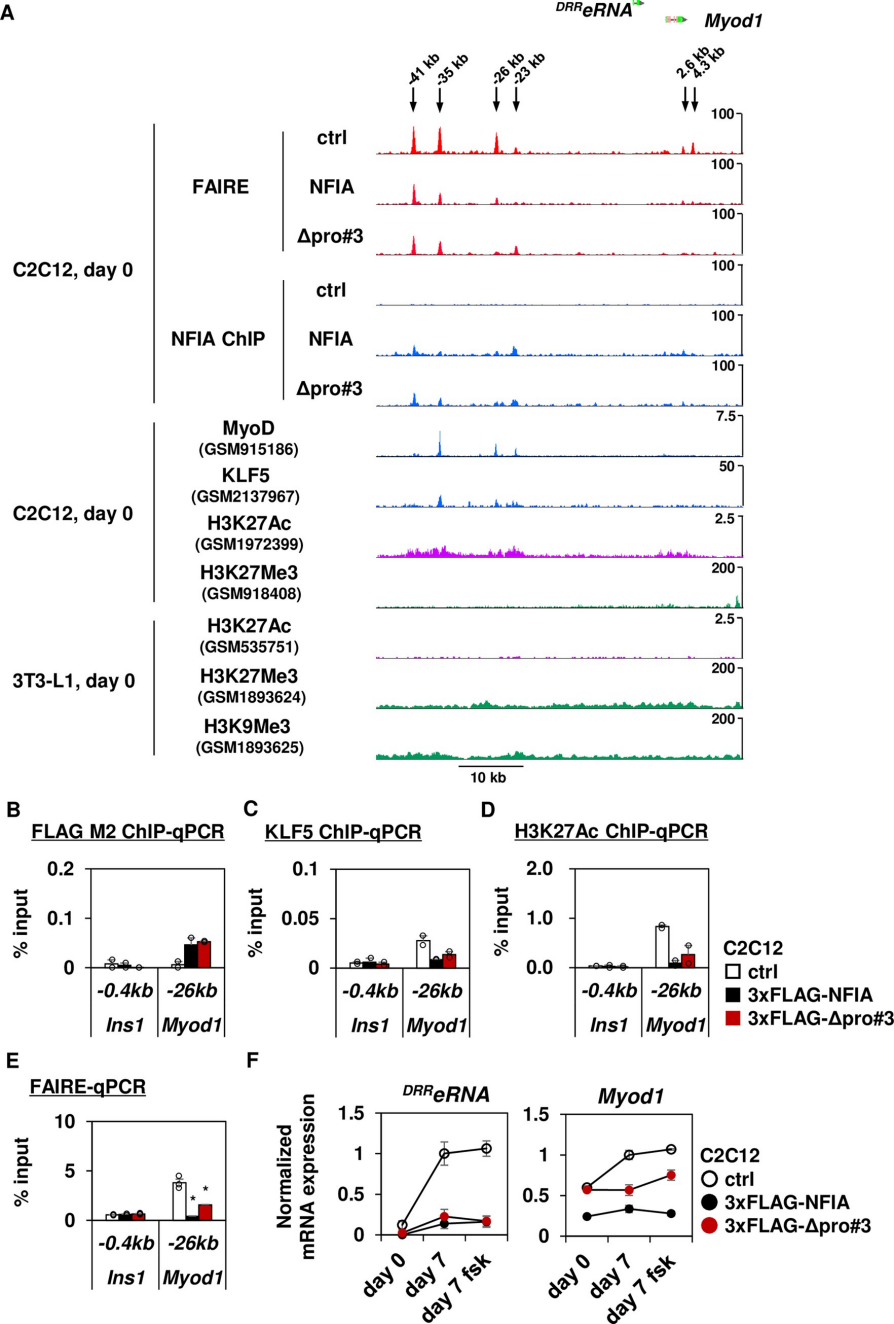
NFIA directly suppresses *Myod1* and ^*DRR*^*eRNA* via competition with KLF5 in terms of enhancer binding, and also suppresses *Myog in trans*. (A) FAIRE-seq and NFIA ChIP-seq tracks in indicated cells at day 0 of differentiation at the *Myod1* locus. ChIP-seq tracks of MyoD, H3K27Ac and H3K27Me3 in C2C12 myoblasts, H3K27Ac, H3K9Me3 and M3K27Me3 in 3T3-L1 preadipocytes were obtained by gene expression omnibus (GEO) dataset as indicated. (B) 3xFLAG-NFIA as well as 3xFLAG-Δpro#3 ChIP-qPCR analysis using the FLAG M2 antibody, of indicated locus. The *Ins1*–0.4kb site is shown as a background site. (mean +/- S.E.M.; N = 2.). (C) KLF5 ChIP-qPCR analysis of indicated locus. The *Ins1*–0.4kb site is shown as a background site. (mean +/- S.E.M.; N = 2.). (D) H3K27Ac ChIP-qPCR analysis of indicated locus. The *Ins1*–0.4kb site is shown as a background site. (mean +/- S.E.M.; N = 2.). (E) FAIRE-qPCR analysis of indicated locus. The *Ins1*–0.4kb site is shown as a background site (mean +/- S.E.M.; N = 3; * *P* <0.05, ** *P* <0.01). The representative result of multiple independent experiments is shown. (F) Normalized mRNA expression during adipocyte differentiation of ^*DRR*^*eRNA* and *Myod1* (mean +/- S.E.M.; N = 3). When indicated, forskolin (fsk) treatment was performed to increase intracellular cyclic AMP levels.

While Δpro#3 mutant almost completely lacked the ability to activate genes up-regulated by full-length NFIA, the mutant could still retain the ability to repress genes down-regulated by full-length NFIA (Fig 4A). Since the gene cluster down-regulated by both full-length NFIA and Δpro#3 mutant was characterized by GO terms related to muscle development and function (cluster E, Fig 4A and 4F), we hypothesized that these genes could be coordinately down-regulated by a few of crucial transcriptional regulators required for myogenesis. In this regard, we observed the binding of both full-length NFIA and Δpro#3 mutant to multiple pre-accessible sites near *Myod1*, which encodes a master transcriptional regulator of myogenesis, MyoD (*Myod1* -41kb, -35kb, -26kb, -23kb, 2.6kb and 4.3kb). Notably, these pre-accessible and NFIA-bound sites overlapped with active histone mark H3K27Ac in C2C12 myoblasts and reciprocally overlapped with repressive histone marks such as H3K9Me3 and H3K27Me3 in 3T3-L1 preadipocytes [12–15], suggesting that during adipogenesis, NFIA binds to functional enhancers required for myogenesis and suppress their activity (Fig 7A). We observed, too, that the binding of NFIA also overlaps with the binding of MyoD itself [14], suggesting that NFIA disrupts the positive feedback loop of *Myod1* expression and its up-regulation by MyoD (Fig 7A). We also observed the overlap of NFIA binding with that of KLF5, a known positive regulator of myogenesis [10]. Strikingly, we found that binding of full-length NFIA as well as the Δpro#3 mutant to the *Myod1* -26kb enhancer resulted in reciprocal repression of the KLF5 binding signal to the same locus (Fig 7B and 7C), suggesting the direct competition of this locus between NFIA and KLF5. Although KLF5 protein expression was down-regulated by introduction of full-length NFIA (S9A Fig), the binding signal of KLF5 to the *Myod1* -26kb enhancer was disproportionally decreased upon introduction of full-length NFIA as well as Δpro#3 mutant. Moreover, the binding of both full-length NFIA and Δpro#3 mutants decreased chromatin accessibility and H3K27Ac signals at the *Myod1* -26kb locus (Fig 7A, 7D and 7E). On the other hand, forced expression of KLF5 resulted in increased KLF5 binding and decreased NFIA binding to the *Myod1* -26kb enhancer, consistent with reciprocal relationship between NFIA and KLF5 at this locus (S9B and S9C Fig). However, we did not observe rescued expression of *Myod1* as well as its target *Myog* upon forced expression of KLF5 (S9D Fig), suggesting that NFIA exerts anti-myogenic effect through multiple pathways and expression of KLF5 alone is not sufficient to rescue the impaired myogenesis. Intriguingly, we observed that a long noncoding RNA *Gm45923* (also known as ^*DRR*^*eRNA*), which is located 4 kb upstream of *Myod1*, was even more severely down-regulated by full-length NFIA and by Δpro#3 mutants than *Myod1* (Fig 7F). The noncoding RNA ^*DRR*^*eRNA* is a muscle specific enhancer RNA transcribed from locus near *Myod1*, being recruited to the *Myog* locus to facilitate cohesion binding to the *Myog* enhancer and activate *Myog* transcription [5]. So down-regulation of ^*DRR*^*eRNA* might account for the fact that *Myog* was more severely down-regulated by full-length NFIA and Δpro#3 mutants than was *Myod1—*even though we did not find significant NFIA binding nor changes in chromatin accessibility near the *Myog* locus (S9E Fig). Collectively, these results suggest that NFIA negatively regulates myogenesis, at least in part via suppression of KLF5 binding to the *Myod1* -26kb enhancer by direct competition, resulting in down-regulation of *Myod1*. And this phenomenon does not require pro#3 domain.

### Pro#3 domain is dispensable for recruiting PPARγ to the brown-fat-specific enhancers in the late phase of differentiation

We previously found that NFIA is able to facilitate the binding of PPARγ to the brown-fat-specific enhancers and activate target gene expression in cells with overexpressed PPARγ [2]. To find whether this ability is retained by Δpro#3 mutant, we introduced either PPARγ alone, PPARγ and full-length NFIA, or PPARγ and Δpro#3 mutant into C2C12 cells and induced adipocyte differentiation. The degree of adipocyte differentiation was comparable throughout the three groups as evaluated by Oil-Red-O staining and by *Fabp4* expression (S10A and S10B Fig). We confirmed that both full-length NFIA and Δpro#3 mutant binds to the enhancer of brown-fat-specific genes such as *Ppargc1a* and *Ucp1* in this model system (S10C Fig). As previously reported, full-length NFIA facilitated the binding of PPARγ to the enhancer of these genes (S10D Fig). Interestingly, Δpro#3 mutant also facilitated the binding of PPARγ. Accordingly, chromatin accessibility of those enhancers was increased by both full-length NFIA and Δpro#3 mutant (S10E Fig). Finally, co-introduction of PPARγ and Δpro#3 mutant significantly increased *Ucp1* and *Ppargc1a* expression—although the degree of increase was small when compared with when PPARγ and full-length NFIA were co-introduced (S10F Fig). Up-regulation of *Ucp1* and *Ppargc1a* was not observed when we co-introduced PPARγ and ΔTAD mutant, suggesting that facilitation of PPARγ is mediated by a distinct functional domain within TAD but outside of the pro#3 domain (S11A–S11C Fig). These results suggest that, although Δpro#3 mutant cannot drive *Pparg* expression in the early phase of adipogenesis, this mutant can still activate the brown-fat-specific enhancers by facilitating the binding of PPARγ to these sites in the late phase of brown adipocyte differentiation.

## Discussion

In this study, we identified the C-terminal 17 amino acid residues of NFIA (aa 493–509)—which we called the pro#3 domain—as a region critical for NFIA to positively regulate *Pparg* expression and adipogenesis. The Δpro#3 mutant almost totally lacked the transcriptional activity. Indeed, introduction of Δpro#3 mutant failed to rescue the impaired beige adipogenesis caused by Cre-mediated NFIA-KO in primary adipocytes. However, transcriptome analysis showed that although Δpro#3 mutant cannot activate genes up-regulated by full-length NFIA, it still can repress genes down-regulated by full-length NFIA. Mechanistically, the ability to penetrate otherwise inaccessible chromatin and increase accessibility underlie the differential effect of full-length NFIA and Δpro#3 mutant, while pro#3 domain is dispensable for negative regulation of chromatin accessibility. Generally, TADs of transcription factors interact with co-regulators such as Mediator complex, histone modification enzymes and SWI/SNF ATP-dependent chromatin remodeling factors. Pro#3 domain might specifically interact with co-activators to activate target gene expression.

We showed, too, that NFIA also possesses a direct role in suppressing myogenesis—at least by repressing the expression of *Myod1* and ^*DRR*^*eRNA* via suppression of KLF5 binding to their enhancer by direct competition, resulting in *Myog* repression *in trans*. Since PPARγ and MyoD have been reported to antagonize each other [6] and we showed here that Δpro#3 mutant retains the ability to repress *Myod1*, ^*DRR*^*eRNA* and *Myog* even though this mutant cannot induce *Pparg* transcription, these results collectively indicate that NFIA suppresses the myogenic gene program through both the PPARγ-dependent and the PPARγ–independent pathways to ensure adipocyte differentiation. Unbiased proteomic analysis of NFIA protein complex to determine the factor(s) responsible for both the positive and negative effect of NFIA on gene regulation constitutes an attractive future direction for study.

Another reasonable approach to understand why NFIA is able to exert an opposing effect on the adipogenic and myogenic gene programs is to explore the possible difference in co-localizing and/or competing transcription factor(s) between loci near adipogenic genes and myogenic genes genome-wide. We previously showed that facilitating the binding of PPARγ and co-localizing with them at the brown-fat-specific enhancers is a key mechanistic feature for NFIA to control the brown fat gene program [2]. And here we showed that the binding of NFIA overlaps with that of MyoD and KLF5 near the *Myod1* enhancer. Besides, *de novo* motif analysis of NFIA binding regions within “Lost by NFIA” sites identified enrichment of SP-1, KLF and AP-1 motifs along with NF-1 motif (Fig 5E). It is possible that NFIA functionally interacts with these candidate co-localizing or competing factors to exert negative effect on the myogenic gene program genome-wide, not only near the *Myod1* locus.

A class of transcription factor called pioneer factor can bind to compacted DNA to displace nucleosome by itself alone, increase chromatin accessibility and allow other transcription factors to bind to the sites [16]. Previously, we showed that NFIA activates the brown-fat-specific enhancers even before differentiation and facilitates the binding of other transcription factors including PPARγ, C/EBPs and EBF2, to control the brown fat gene program [2]. The effect of NFIA binding on the brown-fat-specific enhancers and gene expression is very consistent with the concept of pioneer factors. Further studies are needed to understand the precise molecular mechanism(s) underlying the effect of NFIA on chromatin during brown adipocyte differentiation. Also, our findings about Δpro#3 mutant in the late phase of differentiation (S10 Fig) suggest that NFIA can act in varying ways at the chromatin level to positively regulate brown adipocyte differentiation through pathways both dependent and independent of the pro#3 domain.

**Fig 8:**
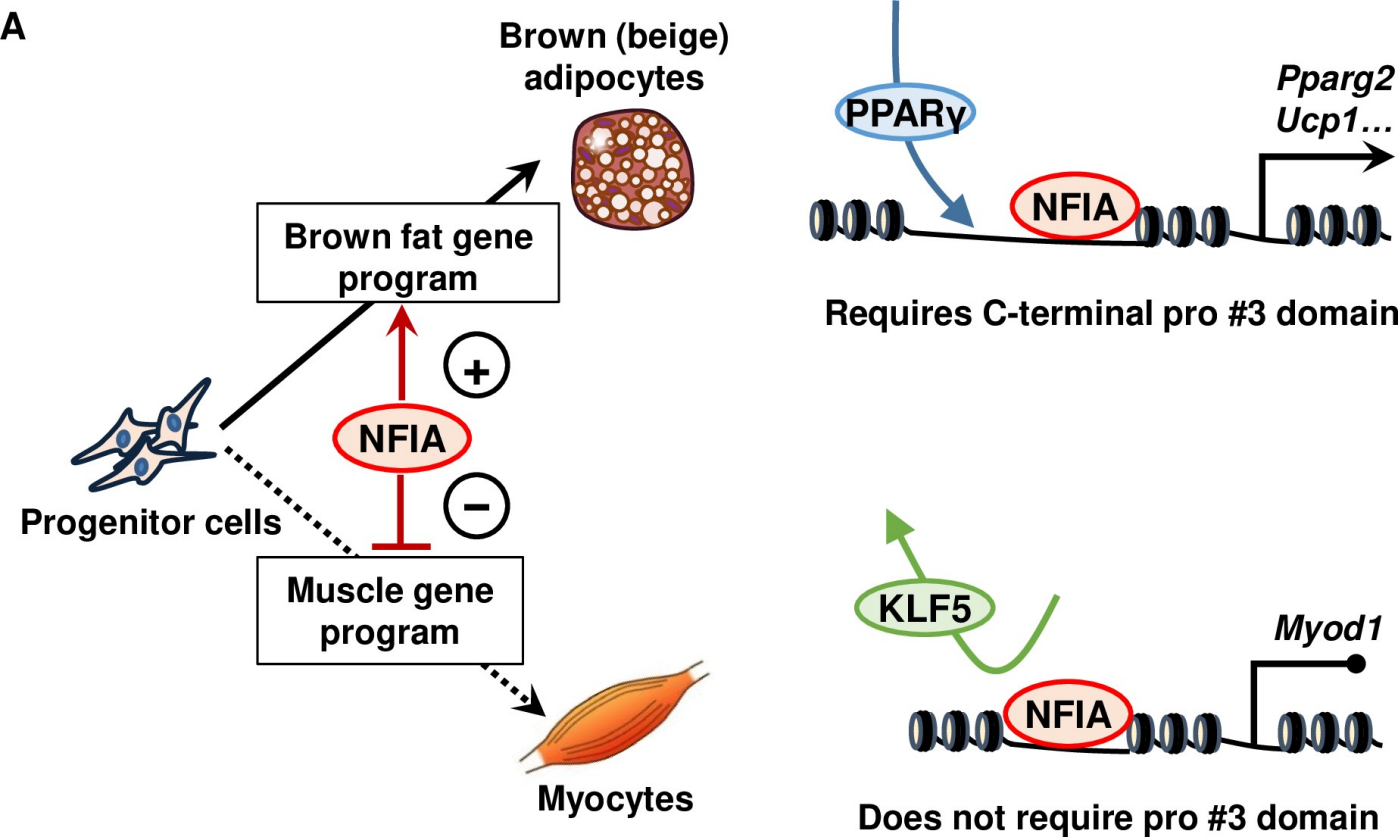
Proposed model of the effect of NFIA on cell fate determination between myocytes and brown/beige adipocytes. (A) NFIA activates *Pparg2* expression through its C-terminal pro#3 domain and co-localizes with PPARγ to control the brown fat gene program. On the other hand, the domain is dispensable for suppression of muscle gene expression through direct suppression of *Myod1* via competition with KLF5 in terms of enhancer binding, in a PPARγ-independent manner.

In conclusion, we found that NFIA activates *Pparg2* expression and adipogenesis through its C-terminal proline rich domain (pro#3 domain). While pro#3 domain is required for the binding of NFIA to otherwise inaccessible chromatin regions to activate *Pparg2* expression and adipogenic gene program, the domain is dispensable for suppression of myoenic gene program through suppression of *Myod1* expression via competition with KLF5 in terms of enhancer binding, in a PPARγ-independent manner (Fig 8). Altogether, these results provide a mechanistic insight into multiple layers of gene regulation by NFIA during brown adipocyte differentiation and an implication for the development of anti-obesity therapy.

## Materials and methods

### Ethics statement

All animal work was approved by Institutional Animal Care and Use Committee (IACUC) of the University of Tokyo (P17-082) and conducted according to the institutional guidelines at The University of Tokyo.

### Generation of *Nfia*^*flox/flox*^ mice

C57BL/6N-A/a ES cells bearing the KO first allele (reporter-tagged insertion with conditional potential) were purchased from EuMMCR (HEPD0646_5_E04), and the cells were injected into mice blastocysts in the RIKEN Center for Biosystems Dynamics Research (Kobe, Japan). Mutant chimeric mice were crossed with C57BL/6J mice to obtain mice with germline transmission. Then the mice were crossed with the Flp mice to remove the Neo cassette in the germline and to obtain *Nfia*^floxflox^ mice. The construct of the floxed *Nfia* allele is shown in S2A Fig.

### Cell culture

C2C12 myoblasts and HEK293 cells were purchased from American Type Culture Cell Collection (ATCC). Stromal vascular fraction (SVF) from inguinal murine white adipose tissue was isolated as reported previously [17] with some optimization for the use of gentleMACS Octo Dissociator with Heaters (Miltenyi Biotec). The cells were then immortalized using retroviral vector expressing SV-40 large T antigen. For adipocyte differentiation of C2C12 expressing NFIA and/or PPARγ, cells were treated as described previously [2]. For adipocyte differentiation of immortalized SVF cells, at the confluence, cells were treated for 48 hours in medium containing 10% FBS, 0.5 mM isobuylmethylxanthine, 125 nM indomethacin, 1 μM dexamethosone, 850 nM insulin, 1 nM T3 and 1 μM rosiglitazone. After 48 hours, cells were switched to medium containing 10% FBS, 850 nM insulin, 1 nM T3 and 1 μM rosiglitazone.

### Retroviral expression system

For gain-of-function experiments, we used the pMXs retroviral expression system as previously described [2]. Retroviral vectors expressing deletion mutant of NFIA were constructed using the KOD mutagenesis kit (TOYOBO) according to the manufacturer’s instructions. A retroviral vector expressing Cre recombinase was a gift from Kai Ge (Addgene plasmid # 34564).

### Luciferase reporter assay

MH100-*Tk*-luciferase reporter vector was co-transfected into HEK293 as well as C2C12 cells along with Renilla luciferase and a vector expressing fusion protein of yeast-derived Gal4 DBD and either full-length or mutant NFIA TAD. Cells were harvested 24 hours after transfection and dual luciferase reporter was assayed by TriStar^2^ LB 942 Modular Multimode Microplate Reader (Berthold). Reporter luciferase activity was normalized to the Renilla luciferase activity.

### ChIP and FAIRE

ChIP was performed as described previously [2] with some modifications. Briefly, samples were treated by nuclear extraction buffer (10 mM Tris-HCl, pH 7.4, 10 mM NaCl, 3 mM MgCl2 and 0.1% IGEPAL CA-630) for 10 minutes and immediately cross-linked with 1% formaldehyde for 7.5 minutes at room temperature. Cross-linking was quenched using 125 mM glycine for 5 minutes. The chromatin was sheared by a probe sonicator (Branson) and was spun at 15,000 rpm for 5 minutes. Antibodies were added for overnight incubation at 4°C. Mixes of Protein A and Protein G Sepharose (GE, for PPARγ andiboty) or Dynabeads Protein A and Protein G (Invitrogen, for FLAG M2, H3K27Ac and KLF5 antibody) were added to samples for 4 hours at 4°C. Subsequent procedures were performed as described previously [2]. The antibodies used were FLAG M2 (Sigma F3165), PPARγ (mix of Santa Cruz Biotechnology, sc-7273, and Perseus Proteomics, A3409A), H3K27Ac (Abcam ab4729) and KLF5 (Abcam ab137676). FAIRE was performed as described previously [2]. ChIP-seq as well as FAIRE-seq libraries were prepared using KAPA hyper prep kit (KAPA Biosystems) according to the manufacturer's instructions.

### RNA expression analysis

Total RNA from cultured cells or tissues was isolated using TRIzol reagent (Invitrogen) and RNeasy Mini columns (QIAGEN). Isolated RNA was reverse-transcribed using ReverTra Ace qPCR RT Master Mix kit (Takara). Real-time quantitative PCR (SYBR green) analysis was performed on QuantStudio 7 Flex Real-Time PCR System (Applied Biosystems). *Rplp0* was used as an internal normalization control. qPCR primers used in this work are listed in S1 Table. For RNA-seq, libraries were prepared using TruSeq Stranded mRNA Library Prep Kit (Illumina) according to the manufacturer's instructions. All the RNA-seq experiments was performed in triplicate.

### Western blotting

Tissues were lysed in radioimmunoprecipitation assay (RIPA) buffer containing 0.1% SDS, 1% NP-40, 0.5% Na deoxycholate, 150 mM NaCl, 50 mM Tris-Cl (pH 8.0), 1 mM EDTA supplemented with protease inhibitor (Roche). Proteins were separated by SDS-PAGE, transferred to nitrocellulose membrane, and detected with the antibodies anti-NFIA (Sigma HPA006111), anti-FLAG M2 (Sigma F3165), KLF5 (Abcam ab137676) and anti-β actin (Sigma A3854).

### CRISPR dCas9-Krab-mediated epigenome editing

Guide RNAs were designed using CRISPOR version 4.92 [18] and cloned into pLV hU6-sgRNA hUbC-dCas9-KRAB-T2a-Puro (Addgene # 71236) using BbsI. Sanger sequencing confirmed guide RNA insertion. Sequences of guide RNAs used in this work are listed in S2 Table. Lentivirus was made by transfection of dCas9-Krab- and guide RNA-expressing plasmid (10 μg), pMD2.G (3.75 μg, addgene #12259) and psPAX2 (7.5 μg, addgene #12260) into HEK293T cells using Lipofectamine 2000. Supernatant was harvested on the next day, and fresh medium was added and incubated for another day. Supernatant was combined and concentrated using PEG-it Virus Precipitation Solution (System Biosciences). 3T3-F442A preadipocytes or NFIA-expressing C2C12 myoblasts were infected with lentivirus along with TransDux MAX Lentivirus Transduction Reagent (System Biosciences). On the next day of infection, cells were selected using puromycin and then used for the subsequent experiment.

### High-throughput sequencing

High-throughput sequencing was performed by using the Illumina Genome Analyzer, Illumina HiSeq 2500 or Illumina MiSeq.

### ChIP-seq and FAIRE-seq data processing

The sequence reads were mapped to UCSC build mm9 (NCBI Build 37) assembly using bowtie2 (Galaxy version 2.3.4) with default parameters [19–22]. Peak calling was performed using MACS2 (Galaxy version 2.1.1.20160309) [23]. Peaks of ChIP-seq and FAIRE-seq were visualized by a GenomeJack browser (version 3.1, Mitsubishi Space Software). Galaxy cistrome [24] was used for genomic region handling. For Venn diagram, note that the sum of the number of peaks in each component may not equal to the number of overall peaks, because a single peak in one sample could overlap with multiple peaks in another sample. *De novo* motif analysis was performed within a 300 bp window around peak centers using MEME-ChIP [25] version 5.0.2 with default parameters. A heat map representation was generated using deepTools2 (Galaxy Version 3.1.2.0.0) [26].

### RNA-seq data processing

The sequence reads were aligned to the mm9 genome assembly using STAR [27] version 2.5.3a with the following parameters:—runThreadN 10,—outSAMtype BAM SortedByCoordinate,—quantMode TranscriptomeSAM GeneCounts,—limitBAMsortRAM 16000000000,—genomeDir MMUC,—readFilesIn Read1.fastq Read2.fastq,—outFilterMultimapNmax 1. Read counts for each gene were derived from the STAR output file named "ReadsPerGene.out.tab" and the matched strand count was used for calculation of fragments per kilobase of exon per million fragment mapped (FPKM). Gene ontology annotation analysis was performed using DAVID [28]. Biological process terms “GO_BP_FAT” were used and GO terms were shown in ascending order of the Benjamini’s adjusted *P* value. A heat map representation was generated using GenePattern online software [29]. For heat map representation, pseudo-count of FPKM 1 was added to all FPKM values to decrease the effect of noise of low-expressed genes, and genes with greater than three fold changes in expression are chosen for subsequent analysis. Row-normalized values were used for hierarchical clustering. Transcript data for evaluating abundance of each *Nfia* isoform were derived from UCSC–mm9 “knownGenemRNA.txt.gz” and EMBL “Mus _musculus.GRCm38.ncrna.fa.gz, Mus_musculus.GRCm38.cdna.all.fa.gz” on 1st July 2019. *Nfia* has seven transcripts for UCSC—uc008tth.2, uc008tti.2, uc008tty.2, uc008tts.2, uc008ttt.2, uc008ttu.2, uc008ttv.2, uc008ttw.2 and uc008ttx.1. and 9 for EMBL—ENSMUST00000152023.7, ENSMUST00000075448.12, ENSMUST00000107062.8, ENSMUST00000052018.11, ENSMUST00000107057.7, ENSMUST00000148930.2, ENSMUST00000092532.12 and ENSMUST00000133011.1. But only two transcripts were perfectly common between the two data (uc008ttx.1 and ENSMUST00000133011.1, uc008ttv.2 and ENSMUST00000092532.12, respectively). And uc008ttvu.2 was almost the same as ENSMUST00000075448.12 (18 base pairs of “GGCCGTGCGGTGCGGTGC” were attached at the 5’ end of uc008ttvu.2.) If the transcript data were mixed in making the index—files to map, the software (Salmon) would produce biased results. So, we made each index for each data UCSC and EMBL separately. Transcripts of uc008tts.2, uc008ttu.2 and uc008tty.2 differ with each exon 1. That of uc008ttv.2 lacks exon 7 of uc008ttu.2. That of uc008ttw.2 lacks exon 7, 9 of uc008ttu.2. Transcript of uc008ttt.2 ends with a few bases attached at exon 6. An uc008ttx.1 starts with alternative exon 1 and ends with longer exon 6. Therefore, a GFF file was generated with known_gene data containing "uc008tty.2“,"uc008ttw.2","uc008ttu.2","uc008ttv.2” and "uc008tts.2”(“uc008ttt.2” and “uc008ttx.1 were excluded). The reference file for the rsem–STAR program was made with that GFF file. The command was “rsem-prepare-reference–gff3 [GFF file]–star mm9.fa [Output REFfile]”. Then we calculated seq-counts using the rsem–STAR program with command “rsem-calculate-expression—star—estimate-rspd—append-names—output-genome-bam [RNA–seq.fastq] [REFfile] [Output Name]”. The estimated isoform counts were collected from all “.isoform.results” files.

### Statistics and reproducibility

Two-tailed student's t-test was performed to determine the statistical significance between two groups unless otherwise specified, with a *P* value of less than 0.05 considered significant. We checked that the data met the assumption of the statistic tests, and variances were similar between the groups being tested.

## Supporting information

S1 Fig The C-terminal region of murine NFIA exhibited substantial amino acid sequence similarity to human NFIC. **(A)** A diagram showing the similarity of amino acid sequence between human NFIC, variant 1 and murine NFIA, variant 2. **(B)** A diagram showing the similarity of amino acid sequences of NFIA between *Homo sapiens*, *Mus musculus*, *Gallus gallus domesticus* and *Danio rerio*.
(TIF)

S2 Fig The pro#3 domain of NFIA is indispensable for driving adipogenesis. **(A)** A diagram showing the KO first allele (reporter-tagged insertion with conditional potential) and floxed *Nfia* allele. Exon 2 of *Nfia* is flanked by two *lox P* sites. **(B)** Control and Cre-expressing immortalized SVF cells derived from *Nfia*^flox/flox^ mice were stained with Oil-Red-O seven days after inducing adipocyte differentiation. **(C)** PCR of genomic DNA using the primer pair that flank the *lox P* sites (mean +/- S.E.M.; N = 3; * *P* <0.05). **(D)** *Nfia* mRNA expression was quantified by RT-qPCRquan at the indicated time course (mean +/- S.E.M.; N = 3; * *P* <0.05, ** *P* <0.01). **(E)** Western blot analysis of NFIA protein expression in control and Cre-expressing cells. β-actin was used as a loading control. **(F-G)** Common adipocyte genes **(F)**, the brown-fat-specific gene *Ppara* **(G)** as well as *Ppargc1a* and *Ucp1* (**H**) were quantified by RT-qPCR at the indicated time course (mean +/- S.E.M.; N = 3; * *P* <0.05, ** *P* <0.01). (I) Expression levels of Cre-recombinase, *Nfia* and *Fabp4* were quantified by RT-qPCR at the indicated time course (mean +/- S.E.M.; N = 3; * *P* <0.05, ** *P* <0.01).
(TIF)

S3 Fig A transcript variant uc008ttu.2 (NM_010905.3, transcript variant 2) is the most abundantly expressed isoform of *Nfia* in adipocytes. **(A)** *Nfia* mRNA expression was quantified by RT-qPCR at the indicated time course (mean +/- S.E.M.; N = 3; * *P* <0.05, ** *P* <0.01). **(B-D)** *Nfib*, *Nfic* and *Nfix* **(B)**, the brown-fat-specific genes *Ppara* and *Ppargc1a* **(C)**, and muscle genes *Myog* and *Myl9* **(D)** were quantified by RT-qPCR at the indicated time course (mean +/- S.E.M.; N = 3; * *P* <0.05, ** *P* <0.01). **(E)** A diagram showing the structure of *Nfia* isoforms obtained from UCSC (seven isoforms) and Refseq (three isoforms). **(F)** qPCR analysis showing the relative abundance of different isoforms of *Nfia* (mean +/- S.E.M.; N = 3). Since expression levels of uc008ttt.2 and uc008ttx.1 were much lower than that of other isoforms, these isoforms were excluded for subsequent RNA-seq analysis. **(G)** RNA-seq analysis showing the abundance of different isoforms of *Nfia* (mean +/- S.E.M.; N = 3).
(TIF)

S4 Fig Hierarchical clustering of genes down- or up-regulated by NFIA-KO and introduction of full-length NFIA as well as the Δpro#3 mutant in NFIA-KO, in adipocytes. (**A**) Venn diagram showing the overlap of genes up-regulated by NFIA-KO, genes down-regulated by 3xFLAG-NFIA in NFIA-KO, and genes down-regulated by 3xFLAG-Δpro#3 mutant. Significantly up- or down-regulated genes were determined by DeSeq2 (3 fold, *P* < 0.05). **(B)** A heat map representation of the hierarchical clustering analysis of genes with greater than three fold changes in expression (N = 1777). Note that pseudo-count of FPKM 1 was added to all FPKM values to decrease the effect of noise of low-expressed genes. Row normalized (FPKM+1) was depicted in heat map after hierarchical clustering. RNA-seq was performed in triplicate for each condition. (**C-I)**, Top GO terms of genes in cluster A-F, as defined in **(B)**.
(TIF)

S5 Fig Changes in the expression patterns of *Nfib*, *Nfic* and *Nfix* upon introduction of full-length NFIA as well as the Δpro#3 mutant. **(A)** RNA-seq analysis showing the abundance of *Nfi* family members in ctrl-, full-length NFIA- as well as Δpro#3 mutant-expressing C2C12 myoblasts at day 7 of differentiation (mean +/- S.E.M.; N = 3). (**B-C**) Normalized mRNA expression during adipocyte differentiation of the brown-fat-specific genes **(B)** and muscle specific genes **(C)** (mean +/- S.E.M.; N = 3). When indicated, forskolin (fsk) treatment was performed to increase intracellular cyclic AMP levels.
(TIF)

S6 Fig Hierarchical clustering of genes down- or up-regulated by introduction of full-length NFIA as well as the Δpro#3 mutant, in C2C12 cells after inducing adipocyte differentiation. **(A)** A heat map representation of the hierarchical clustering analysis of genes with greater than three fold changes in expression (N = 1628). Note that pseudo-count of FPKM 1 was added to all FPKM values to decrease the effect of noise of low-expressed genes. Row normalized (FPKM+1) was depicted in heat map after hierarchical clustering. RNA-seq was performed in triplicate for each condition. (**B-G)**, Top GO terms of genes in cluster A-F, as defined in **(A)**.
(TIF)

S7 Fig Direct binding of full-length NFIA to the *Pparg2* promoter as well as the *Pparg2 77kb* enhancer is indispensable for NFIA-driven adipogenesis. **(A)** Motif analysis within full-length NFIA and Δpro#3 mutant binding sites at day 0 of differentiation. **(B)** qPCR analysis of indicated genes in C2C12 cells with lentivirally introduced dCas9-Krab and indicated guide RNA, along with retrovirally introduced either control, full-length NFIA or the Δpro#3 mutant (mean +/- S.E.M.; N = 3). **(C)** qPCR analysis of indicated genes in 3T3-F442A adipocytes with lentivirally introduced dCas9-Krab and indicated guide RNA (mean +/- S.E.M.; N = 3).
(TIF)

S8 Fig Native chromatin context is required for the transcriptional activity of the *Pparg2* 77kb enhancer. (A) Transcriptional of activity of the *Pparg2* 77kb enhancer, when co-introduced with indicated expression vectors into HEK293 cells, were examined by luciferase reporter assay. (mean +/- S.E.M.; N = 4; ** *P* <0.01). *Fabp4*–5.4kb enhancer is used as a positive control regarding PPARγ-dependent transcriptional activity. When indicated, 1 μM rosiglitazone (Rosi) was added into the medium (**B**) FAIRE-seq and NFIA ChIP-seq tracks at day 0 of differentiation at the *Cebpa* locus.
(TIF)

S9 Fig NFIA directly suppresses *Myod1* and ^*DRR*^*eRNA* and also suppresses *Myog in trans*. **(A)** Western blot analysis of NFIA and KLF5 protein expression in indicated cells. β-actin was used as a loading control. **(B)** KLF5 ChIP-qPCR analysis of indicated locus. The *Ins1*–0.4kb site is shown as a background site. (mean +/- S.E.M.; N = 2.). **(C)** NFIA ChIP-qPCR analysis of indicated locus. The *Ins1*–0.4kb site is shown as a background site. (mean +/- S.E.M.; N = 2.). (**D**) *Myod1* and *Myog* were quantified by RT-qPCR at the indicated time course (mean +/- S.E.M.; N = 3). **(E)** FAIRE-seq and NFIA ChIP-seq tracks at day 0 of differentiation at the *Myog* locus.
(TIF)

S10 Fig Pro#3 domain is dispensable for recruiting PPARγ to the brown-fat-specific enhancers in the late phase of differentiation. **(A)** C2C12 myoblasts expressing only PPARγ, both PPARγ and full-length NFIA, and both PPARγ and Δpro#3 mutant were stained with Oil-Red-O seven days after inducing adipocyte differentiation. **(B)** Normalized mRNA expression of *Fabp4* were quantified by RT-qPCR at the indicated time course (mean +/- S.E.M.). **(C)** NFIA ChIP-qPCR analysis of indicated loci. *Ppargc1a* -97kb and *Ucp1* 9.5kb are background sites (mean +/- S.E.M.; N = 2). The representative result of multiple independent experiments is shown. **(D)** PPARγ ChIP-qPCR analysis of indicated loci. *Ppargc1a* -97kb and *Ucp1* 9.5kb are background sites (mean +/- S.E.M.; N = 2). The representative result of multiple independent experiments is shown. **(E)** FAIRE-qPCR analysis of indicated loci. *Ppargc1a* -97kb and *Ucp1* 9.5kb are background sites (mean +/- S.E.M.; N = 3; * *P* <0.05, ** *P* <0.01). **(F)** Normalized mRNA expression of *Ppargc1* and *Ucp1* were quantified by RT-qPCR at the indicated time course (mean +/- S.E.M.; N = 3; * *P* <0.05, ** *P* <0.01). When indicated, forskolin (fsk) treatment was performed to increase intracellular cyclic AMP levels.
(TIF)

S11 Fig ΔTAD mutant cannot induce expression of the brown-fat-specific genes even in the presence of PPARγ. **(A)** C2C12 myoblasts expressing only PPARγ, both PPARγ and full-length NFIA, and both PPARγ and ΔTAD mutant, were stained with Oil-Red-O seven days after inducing adipocyte differentiation. **(B-C)**, Normalized mRNA expression of *Fabp4* **(B)**, *Ppargc1* and *Ucp1* **(C)** were quantified by RT-qPCR at the indicated time course (mean +/- S.E.M.; N = 3; * *P* <0.05, ** *P* <0.01). When indicated, forskolin (fsk) treatment was performed to increase intracellular cyclic AMP levels.
(TIF)

S1 Table A list of Primers used for qPCR analysis. (XLSX)

S2 Table A list of guide RNA sequences used for CRISPR dCas9-Krab experiments. (XLSX)

S3 Table All numerical data underlying graphs shown in this work. (XLSX)
