# Supplementary figures and images for: NFIA differentially controls adipogenic and myogenic gene program through distinct pathways to ensure brown and beige adipocyte differentiation
Source: PLoS Genet. 2020 Sep 29;16(9):e1009044. doi: 10.1371/journal.pgen.1009044 (PMC7546476; doi:10.1371/journal.pgen.1009044)

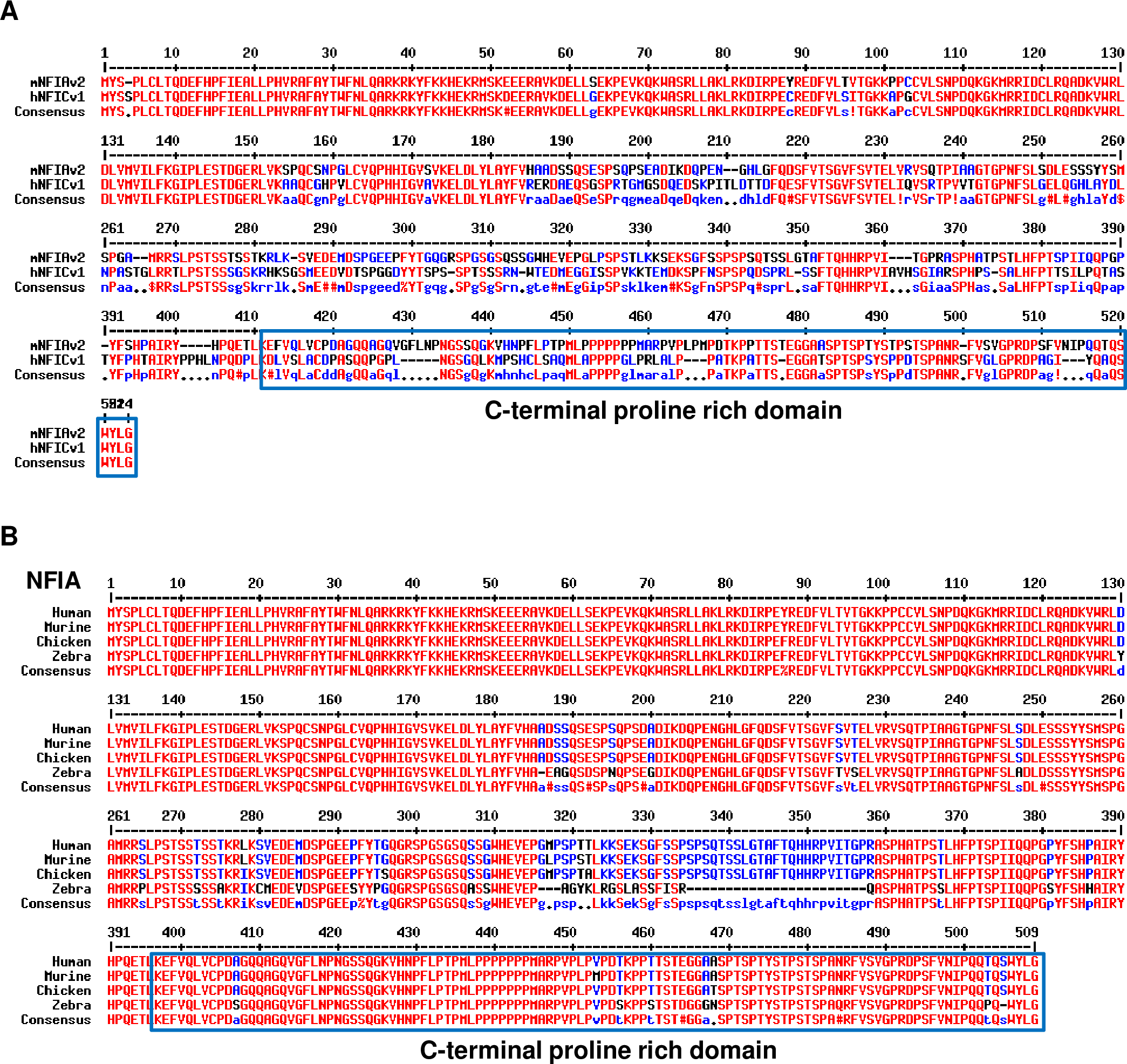

Supplement: S1 Fig — (A) A diagram showing the similarity of amino acid sequence between human NFIC, variant 1 and murine NFIA, variant 2. (B) A diagram showing the similarity of amino acid sequences of NFIA between Homo sapiens, Mus musculus, Gallus gallus domesticus and Danio rerio. (TIF) [file pgen.1009044.s001.tif]

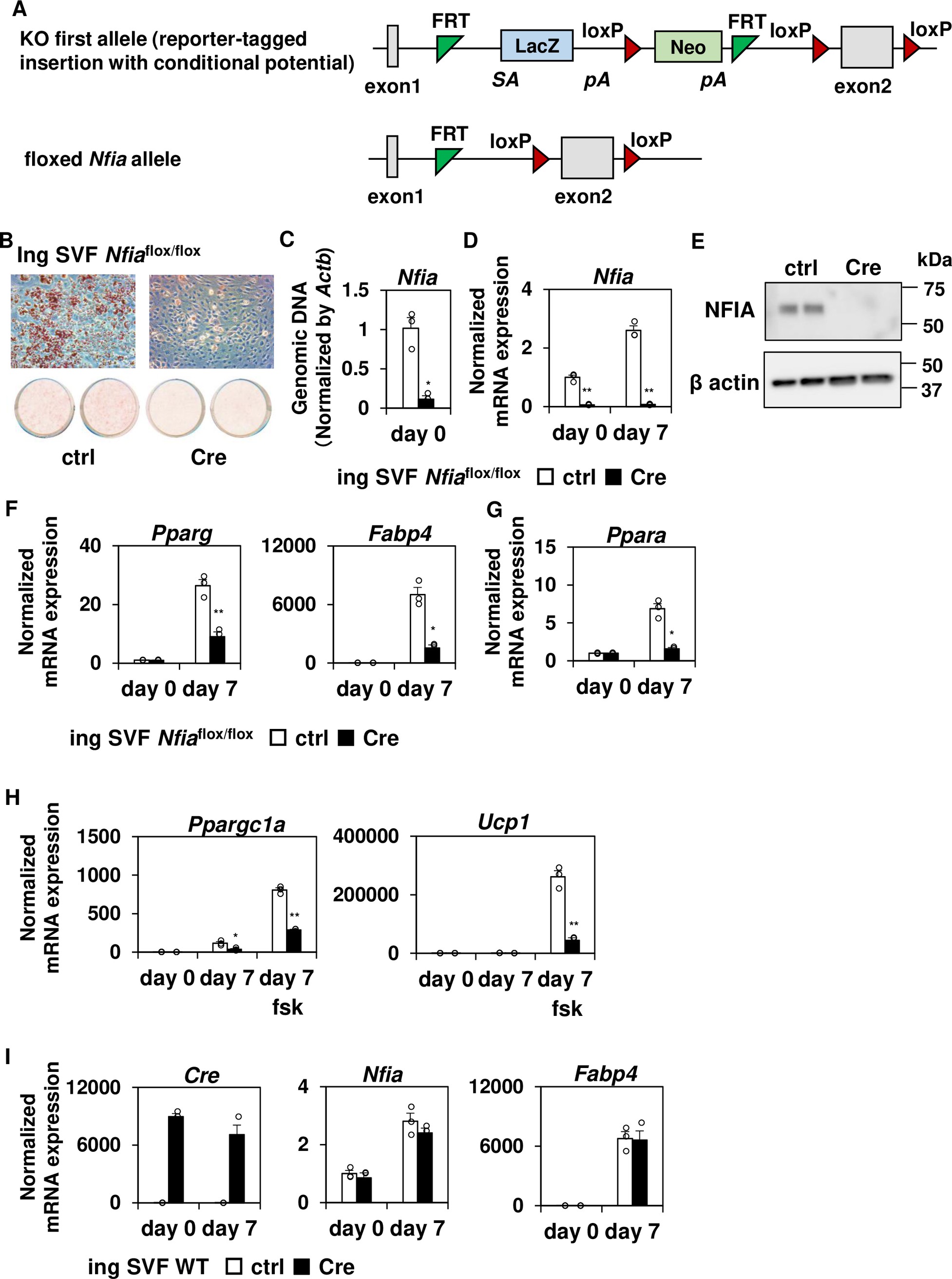

Supplement: S2 Fig — (A) A diagram showing the KO first allele (reporter-tagged insertion with conditional potential) and floxed Nfia allele. Exon 2 of Nfia is flanked by two lox P sites. (B) Control and Cre-expressing immortalized SVF cells derived from Nfiaflox/flox mice were stained with Oil-Red-O seven days after inducing adipocyte differentiation. (C) PCR of genomic DNA using the primer pair that flank the lox P sites (mean +/- S.E.M.; N = 3; * P <0.05). (D) Nfia mRNA expression was quantified by RT-qPCRquan at the indicated time course (mean +/- S.E.M.; N = 3; * P <0.05, ** P <0.01). (E) Western blot analysis of NFIA protein expression in control and Cre-expressing cells. β-actin was used as a loading control. (F-G) Common adipocyte genes (F), the brown-fat-specific gene Ppara (G) as well as Ppargc1a and Ucp1 (H) were quantified by RT-qPCR at the indicated time course (mean +/- S.E.M.; N = 3; * P <0.05, ** P <0.01). (I) Expression levels of Cre-recombinase, Nfia and Fabp4 were quantified by RT-qPCR at the indicated time course (mean +/- S.E.M.; N = 3; * P <0.05, ** P <0.01). (TIF) [file pgen.1009044.s002.tif]

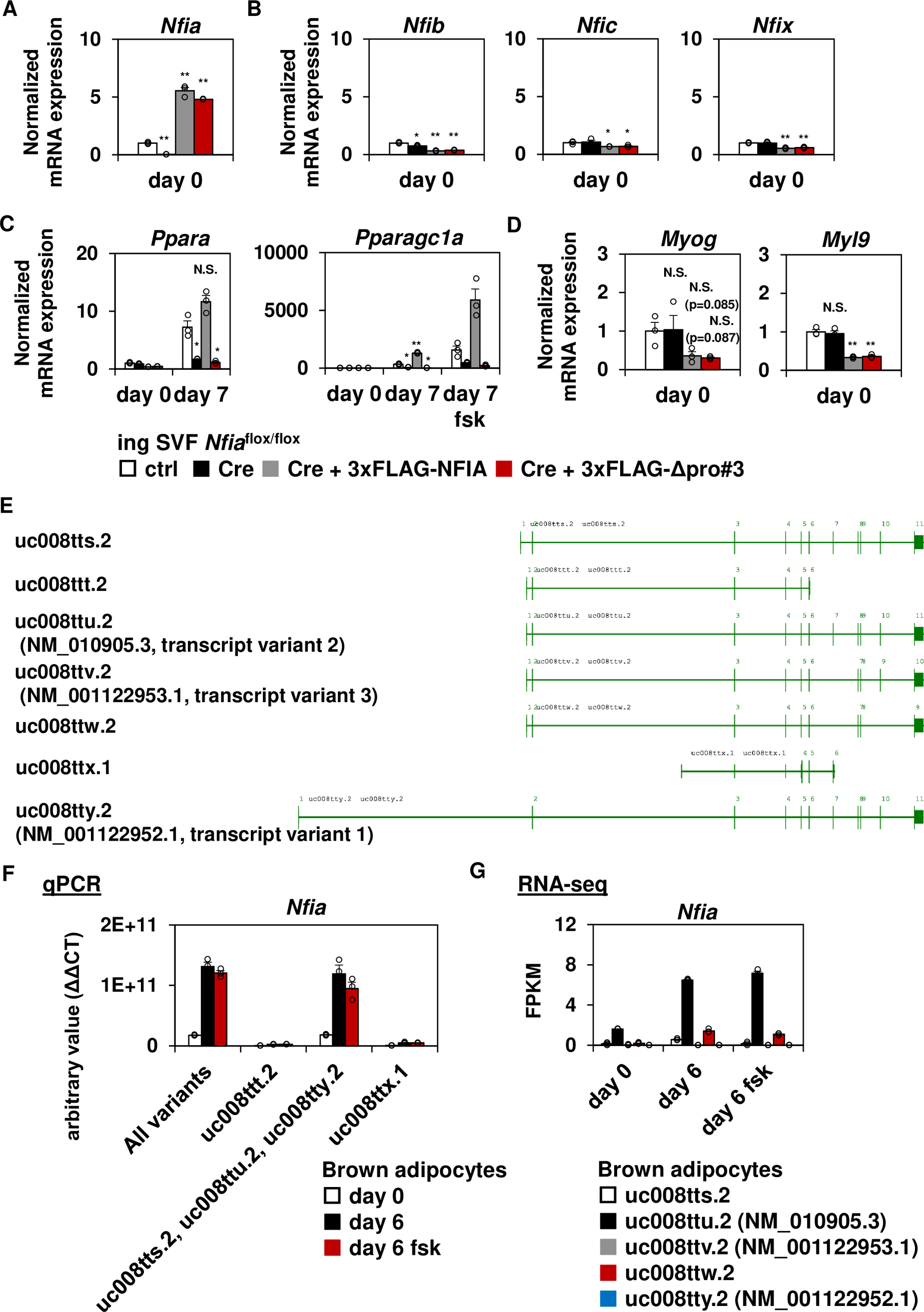

Supplement: S3 Fig — (A) Nfia mRNA expression was quantified by RT-qPCR at the indicated time course (mean +/- S.E.M.; N = 3; * P <0.05, ** P <0.01). (B-D) Nfib, Nfic and Nfix (B), the brown-fat-specific genes Ppara and Ppargc1a (C), and muscle genes Myog and Myl9 (D) were quantified by RT-qPCR at the indicated time course (mean +/- S.E.M.; N = 3; * P <0.05, ** P <0.01). (E) A diagram showing the structure of Nfia isoforms obtained from UCSC (seven isoforms) and Refseq (three isoforms). (F) qPCR analysis showing the relative abundance of different isoforms of Nfia (mean +/- S.E.M.; N = 3). Since expression levels of uc008ttt.2 and uc008ttx.1 were much lower than that of other isoforms, these isoforms were excluded for subsequent RNA-seq analysis. (G) RNA-seq analysis showing the abundance of different isoforms of Nfia (mean +/- S.E.M.; N = 3). (TIF) [file pgen.1009044.s003.tif]

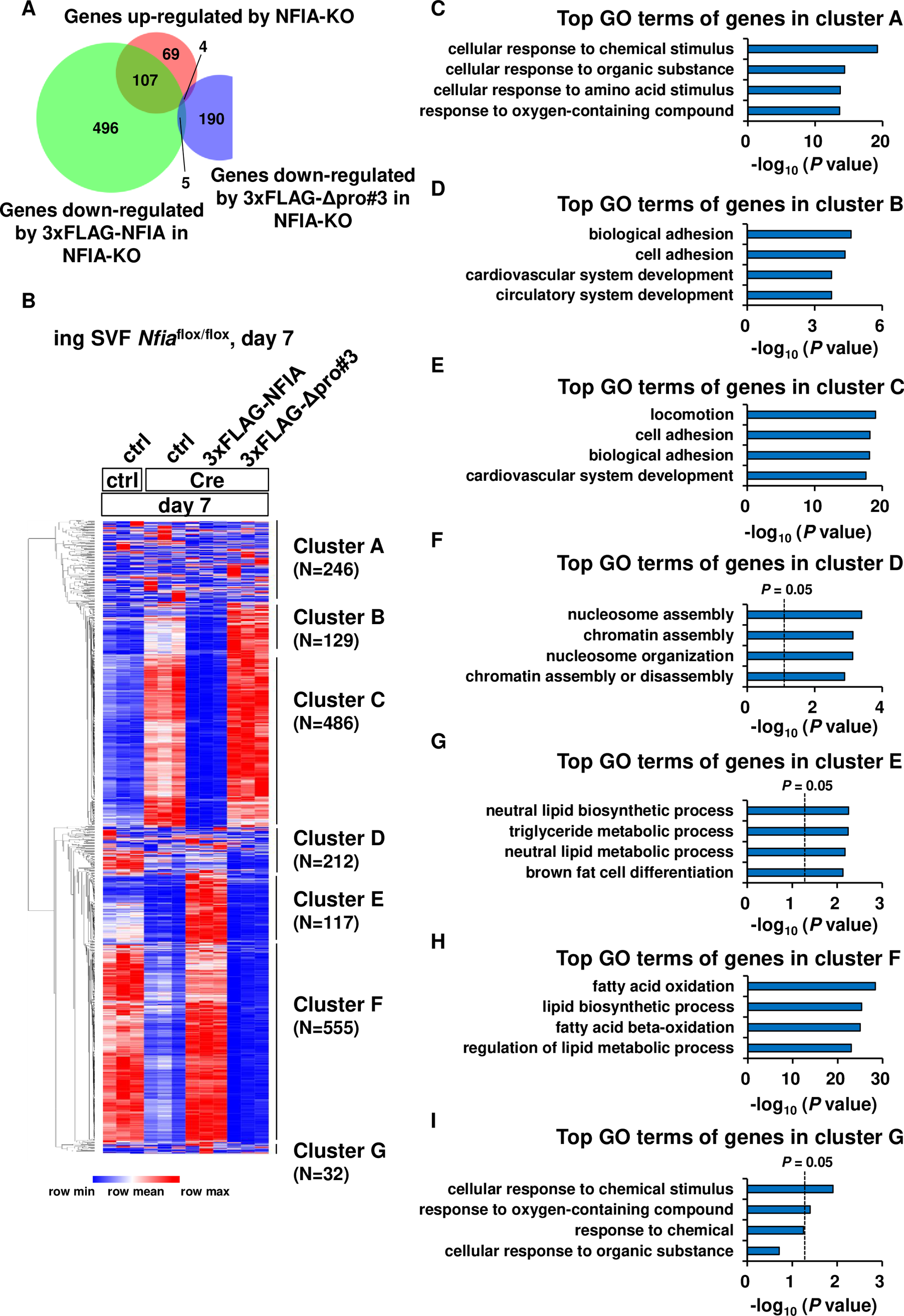

Supplement: S4 Fig — (A) Venn diagram showing the overlap of genes up-regulated by NFIA-KO, genes down-regulated by 3xFLAG-NFIA in NFIA-KO, and genes down-regulated by 3xFLAG-Δpro#3 mutant. Significantly up- or down-regulated genes were determined by DeSeq2 (3 fold, P < 0.05). (B) A heat map representation of the hierarchical clustering analysis of genes with greater than three fold changes in expression (N = 1777). Note that pseudo-count of FPKM 1 was added to all FPKM values to decrease the effect of noise of low-expressed genes. Row normalized (FPKM+1) was depicted in heat map after hierarchical clustering. RNA-seq was performed in triplicate for each condition. (C-I), Top GO terms of genes in cluster A-F, as defined in (B). (TIF) [file pgen.1009044.s004.tif]

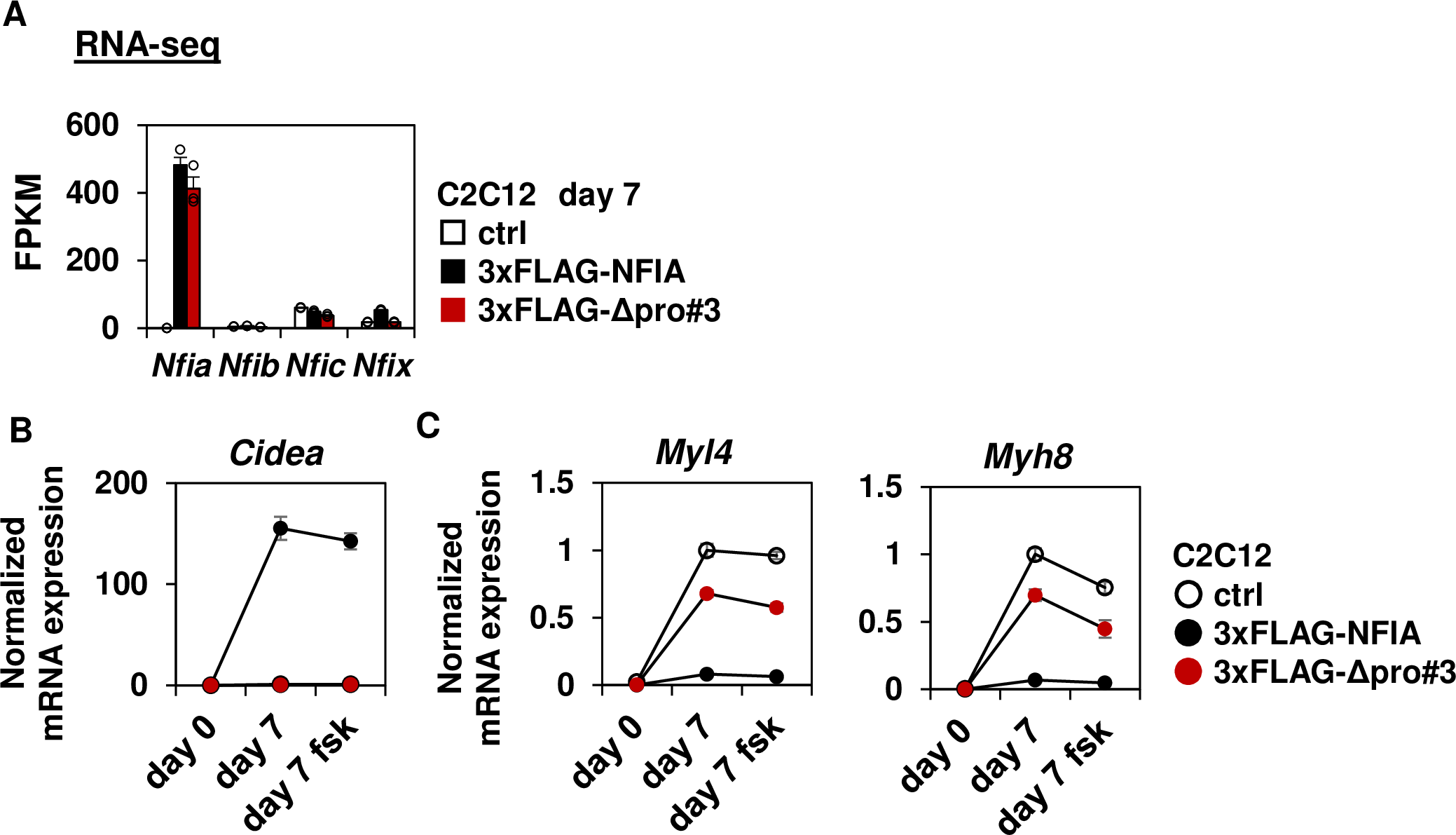

Supplement: S5 Fig — (A) RNA-seq analysis showing the abundance of Nfi family members in ctrl-, full-length NFIA- as well as Δpro#3 mutant-expressing C2C12 myoblasts at day 7 of differentiation (mean +/- S.E.M.; N = 3). (B-C) Normalized mRNA expression during adipocyte differentiation of the brown-fat-specific genes (B) and muscle specific genes (C) (mean +/- S.E.M.; N = 3). When indicated, forskolin (fsk) treatment was performed to increase intracellular cyclic AMP levels. (TIF) [file pgen.1009044.s005.tif]

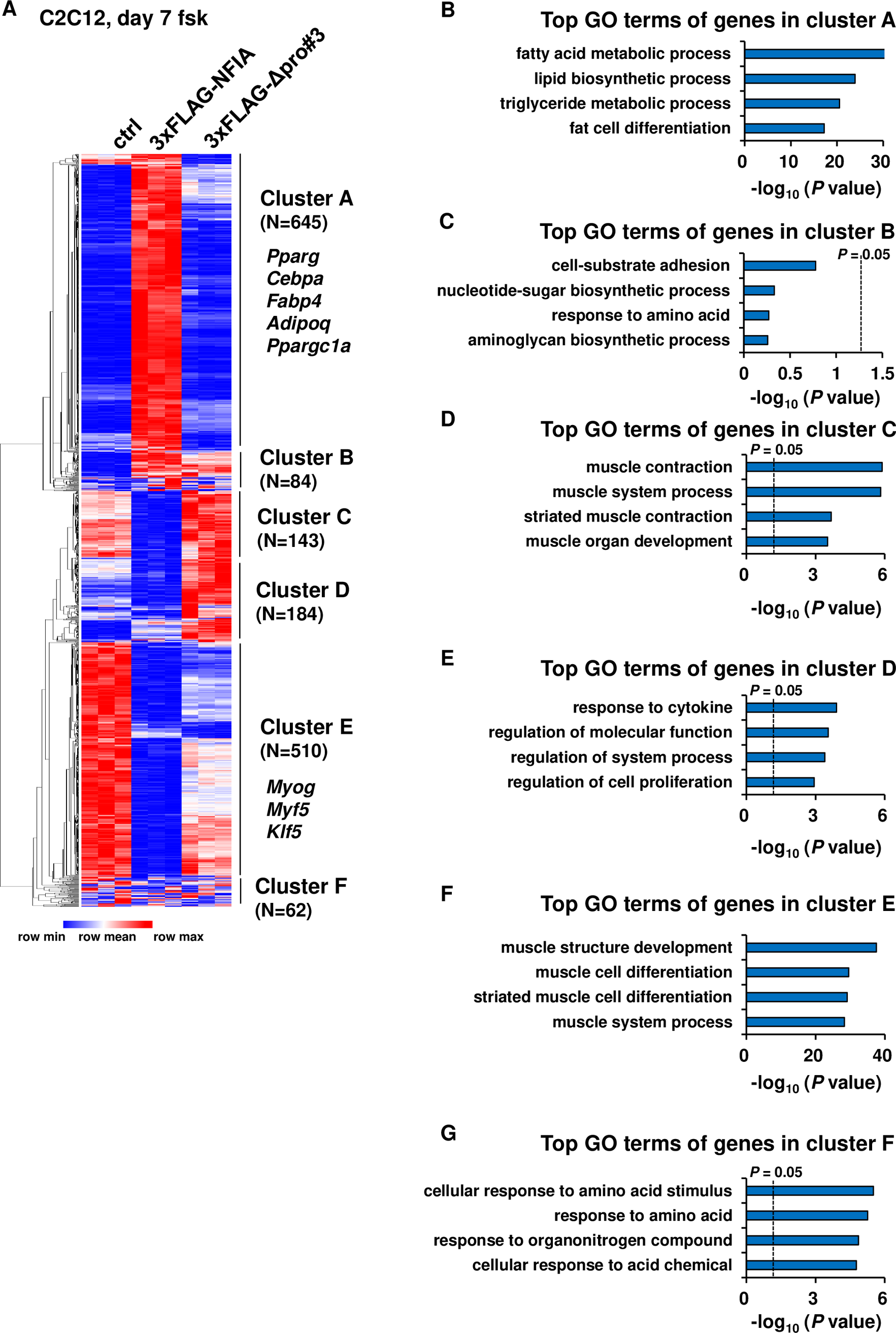

Supplement: S6 Fig — (A) A heat map representation of the hierarchical clustering analysis of genes with greater than three fold changes in expression (N = 1628). Note that pseudo-count of FPKM 1 was added to all FPKM values to decrease the effect of noise of low-expressed genes. Row normalized (FPKM+1) was depicted in heat map after hierarchical clustering. RNA-seq was performed in triplicate for each condition. (B-G), Top GO terms of genes in cluster A-F, as defined in (A). (TIF) [file pgen.1009044.s006.tif]

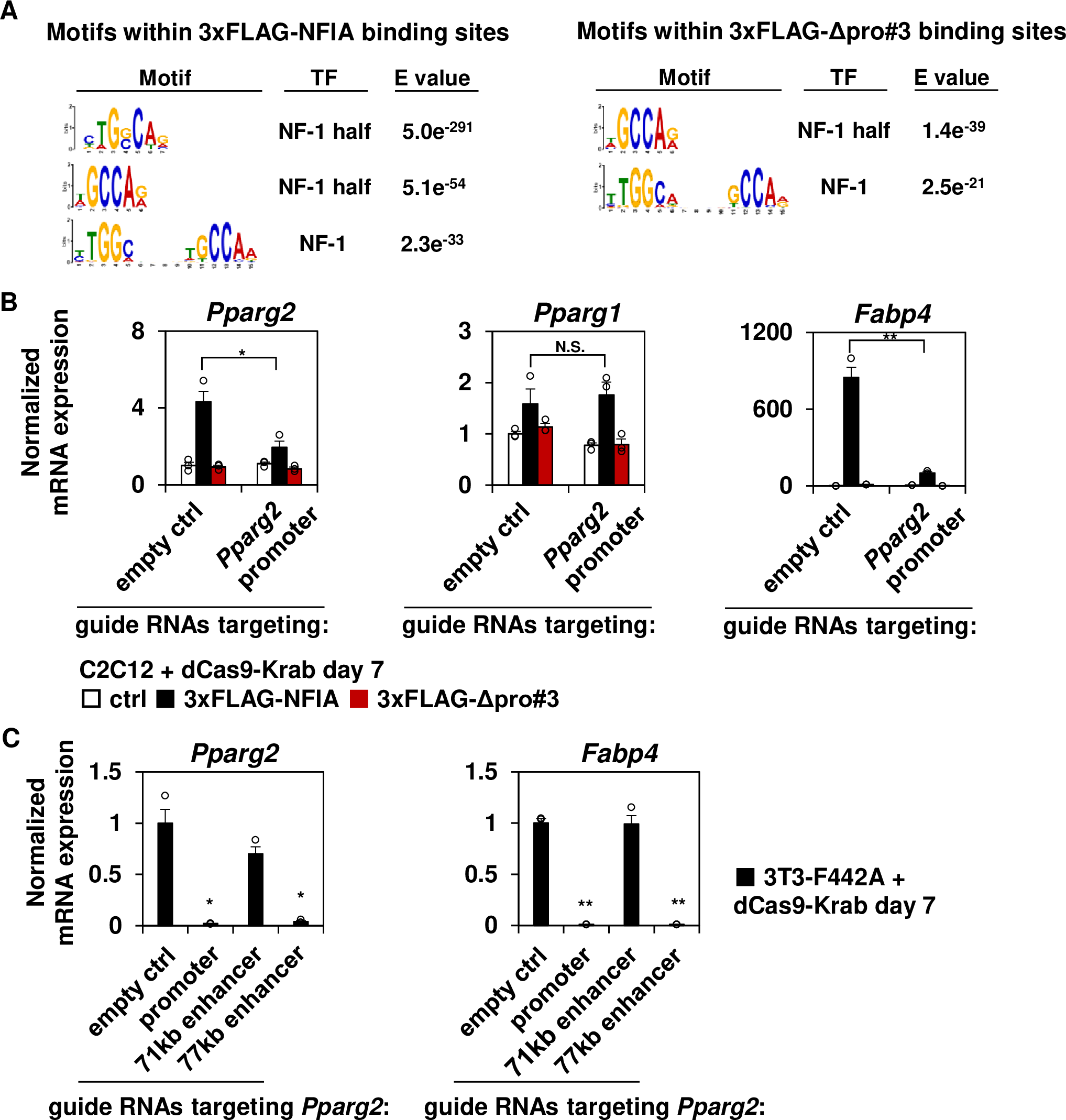

Supplement: S7 Fig — (A) Motif analysis within full-length NFIA and Δpro#3 mutant binding sites at day 0 of differentiation. (B) qPCR analysis of indicated genes in C2C12 cells with lentivirally introduced dCas9-Krab and indicated guide RNA, along with retrovirally introduced either control, full-length NFIA or the Δpro#3 mutant (mean +/- S.E.M.; N = 3). (C) qPCR analysis of indicated genes in 3T3-F442A adipocytes with lentivirally introduced dCas9-Krab and indicated guide RNA (mean +/- S.E.M.; N = 3). (TIF) [file pgen.1009044.s007.tif]

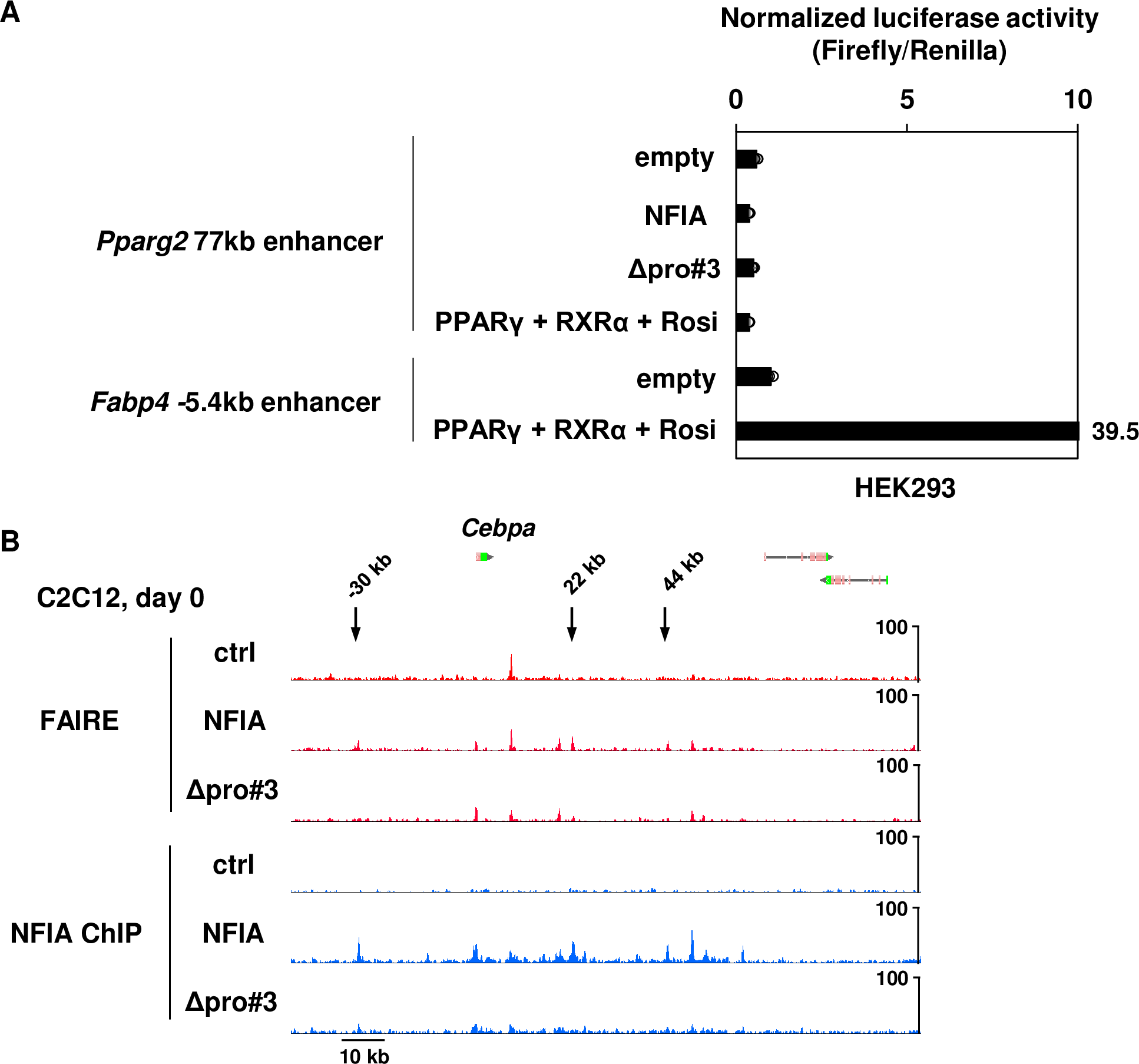

Supplement: S8 Fig — (A) Transcriptional of activity of the Pparg2 77kb enhancer, when co-introduced with indicated expression vectors into HEK293 cells, were examined by luciferase reporter assay. (mean +/- S.E.M.; N = 4; ** P <0.01). Fabp4–5.4kb enhancer is used as a positive control regarding PPARγ-dependent transcriptional activity. When indicated, 1 μM rosiglitazone (Rosi) was added into the medium (B) FAIRE-seq and NFIA ChIP-seq tracks at day 0 of differentiation at the Cebpa locus. (TIF) [file pgen.1009044.s008.tif]

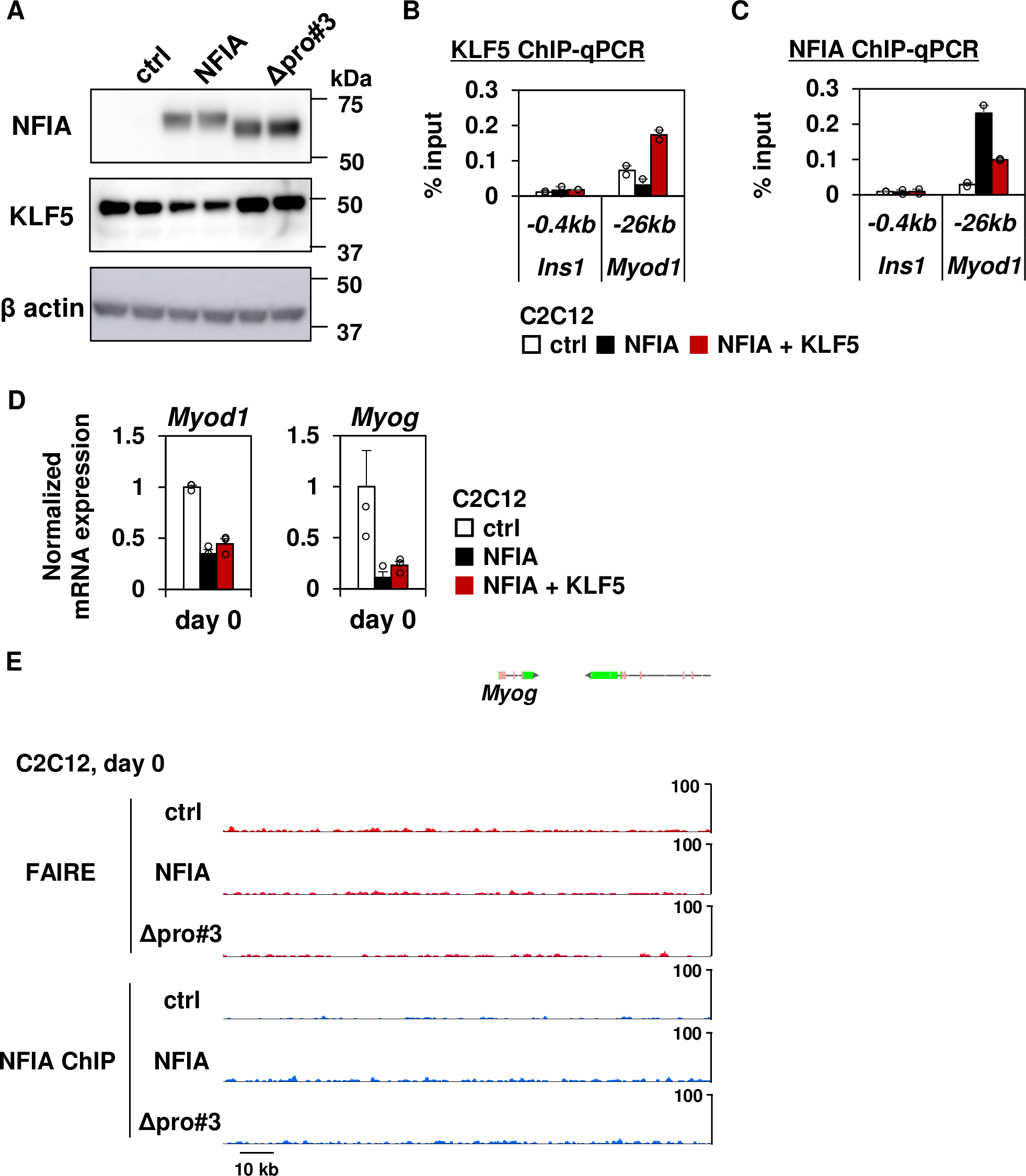

Supplement: S9 Fig — (A) Western blot analysis of NFIA and KLF5 protein expression in indicated cells. β-actin was used as a loading control. (B) KLF5 ChIP-qPCR analysis of indicated locus. The Ins1–0.4kb site is shown as a background site. (mean +/- S.E.M.; N = 2.). (C) NFIA ChIP-qPCR analysis of indicated locus. The Ins1–0.4kb site is shown as a background site. (mean +/- S.E.M.; N = 2.). (D) Myod1 and Myog were quantified by RT-qPCR at the indicated time course (mean +/- S.E.M.; N = 3). (E) FAIRE-seq and NFIA ChIP-seq tracks at day 0 of differentiation at the Myog locus. (TIF) [file pgen.1009044.s009.tif]

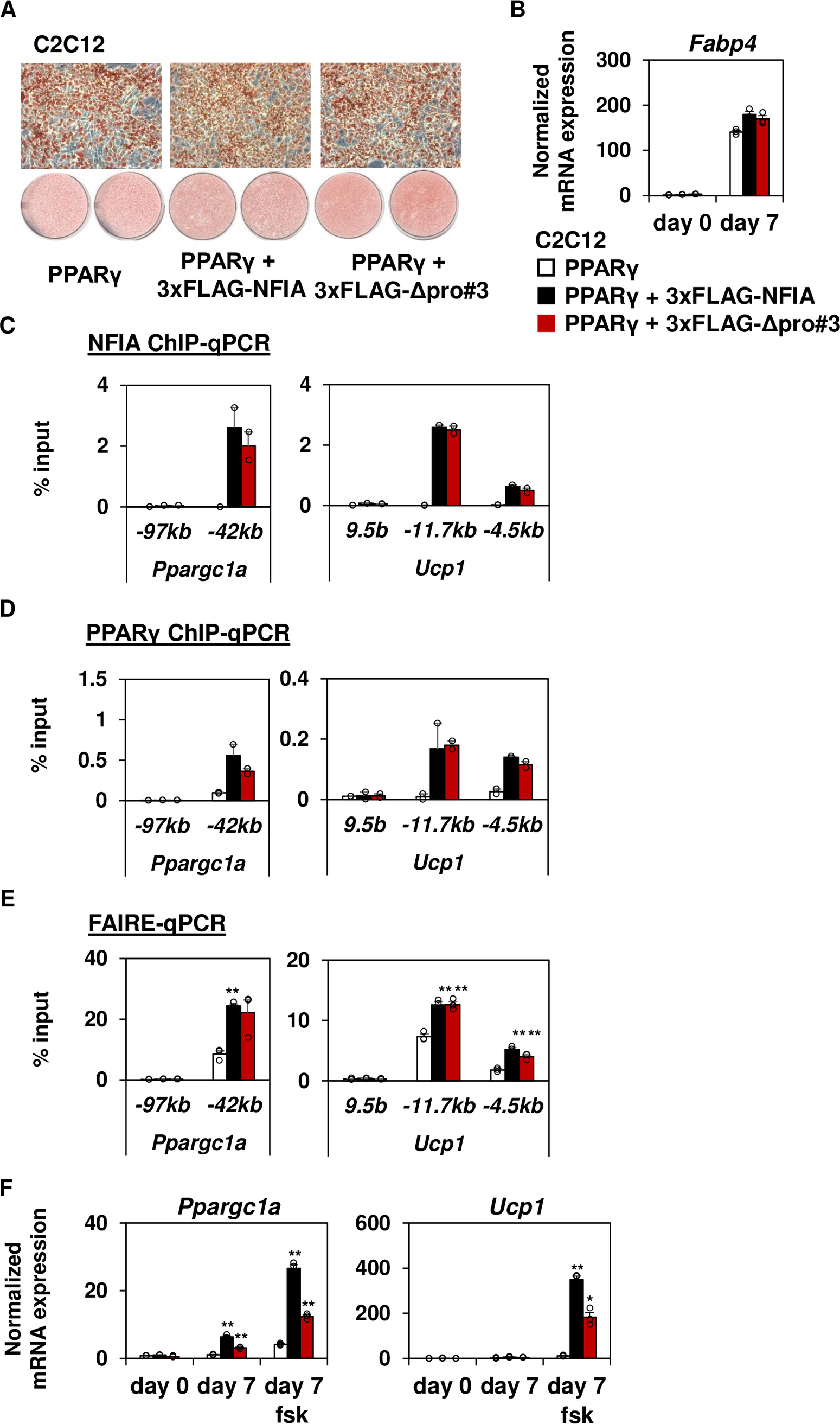

Supplement: S10 Fig — (A) C2C12 myoblasts expressing only PPARγ, both PPARγ and full-length NFIA, and both PPARγ and Δpro#3 mutant were stained with Oil-Red-O seven days after inducing adipocyte differentiation. (B) Normalized mRNA expression of Fabp4 were quantified by RT-qPCR at the indicated time course (mean +/- S.E.M.). (C) NFIA ChIP-qPCR analysis of indicated loci. Ppargc1a -97kb and Ucp1 9.5kb are background sites (mean +/- S.E.M.; N = 2). The representative result of multiple independent experiments is shown. (D) PPARγ ChIP-qPCR analysis of indicated loci. Ppargc1a -97kb and Ucp1 9.5kb are background sites (mean +/- S.E.M.; N = 2). The representative result of multiple independent experiments is shown. (E) FAIRE-qPCR analysis of indicated loci. Ppargc1a -97kb and Ucp1 9.5kb are background sites (mean +/- S.E.M.; N = 3; * P <0.05, ** P <0.01). (F) Normalized mRNA expression of Ppargc1 and Ucp1 were quantified by RT-qPCR at the indicated time course (mean +/- S.E.M.; N = 3; * P <0.05, ** P <0.01). When indicated, forskolin (fsk) treatment was performed to increase intracellular cyclic AMP levels. (TIF) [file pgen.1009044.s010.tif]

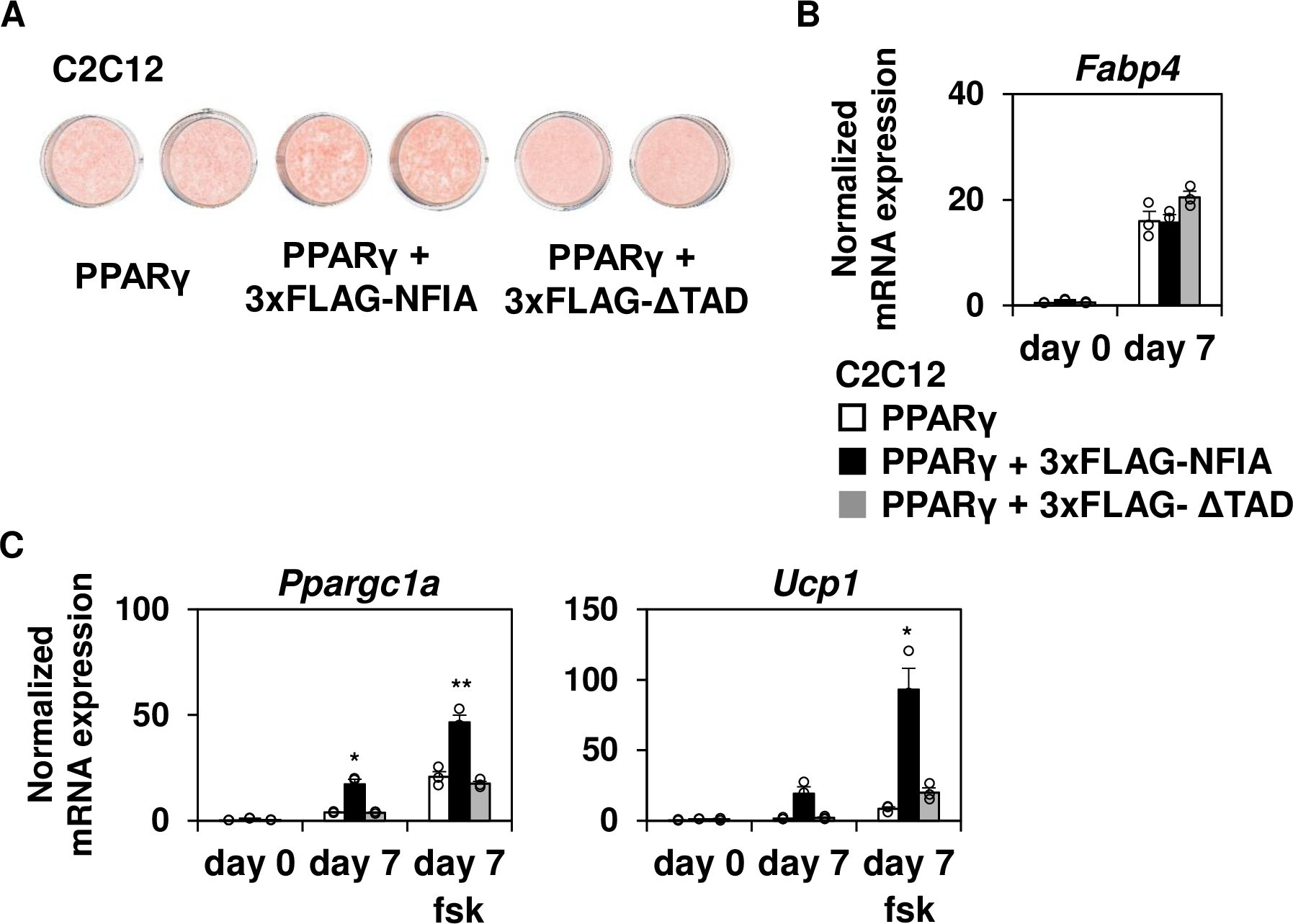

Supplement: S11 Fig — (A) C2C12 myoblasts expressing only PPARγ, both PPARγ and full-length NFIA, and both PPARγ and ΔTAD mutant, were stained with Oil-Red-O seven days after inducing adipocyte differentiation. (B-C), Normalized mRNA expression of Fabp4 (B), Ppargc1 and Ucp1 (C) were quantified by RT-qPCR at the indicated time course (mean +/- S.E.M.; N = 3; * P <0.05, ** P <0.01). When indicated, forskolin (fsk) treatment was performed to increase intracellular cyclic AMP levels. (TIF) [file pgen.1009044.s011.tif]
